# Unlocking the secrets of the immunopeptidome: MHC molecules, ncRNA peptides, and vesicles in immune response

**DOI:** 10.3389/fimmu.2025.1540431

**Published:** 2025-01-29

**Authors:** Arpita Balakrishnan, Gabriela Winiarek, Olga Hołówka, Jakub Godlewski, Agnieszka Bronisz

**Affiliations:** ^1^ Tumor Microenvironment Laboratory, Mossakowski Medical Research Institute, Polish Academy of Sciences, Warsaw, Poland; ^2^ Translational Medicine Doctoral School, Centre of Postgraduate Medical Education, Warsaw, Poland; ^3^ Department of NeuroOncology, Mossakowski Medical Research Institute, Polish Academy of Sciences, Warsaw, Poland

**Keywords:** immunopeptidome, MHC molecules, ncRNA-Derived peptides, extracellular vesicles (EVs), cancer immunotherapy

## Abstract

The immunopeptidome, a diverse set of peptides presented by Major Histocompatibility Complex (MHC) molecules, is a critical component of immune recognition and response. This review article delves into the mechanisms of peptide presentation by MHC molecules, particularly emphasizing the roles of ncRNA-derived peptides and extracellular vesicles (EVs) in shaping the immunopeptidome landscape. We explore established and emerging insights into MHC molecule interactions with peptides, including the dynamics of peptide loading, transport, and the influence of cellular and genetic variations. The article highlights novel research on non-coding RNA (ncRNA)-derived peptides, which challenge conventional views of antigen processing and presentation and the role of EVs in transporting these peptides, thereby modulating immune responses at remote body sites. This novel research not only challenges conventional views but also opens up new avenues for understanding immune responses. Furthermore, we discuss the implications of these mechanisms in developing therapeutic strategies, particularly for cancer immunotherapy. By conducting a comprehensive analysis of current literature and advanced methodologies in immunopeptidomics, this review aims to deepen the understanding of the complex interplay between MHC peptide presentation and the immune system, offering new perspectives on potential diagnostic and therapeutic applications. Additionally, the interactions between ncRNA-derived peptides and EVs provide a mechanism for the enhanced surface presentation of these peptides and highlight a novel pathway for their systemic distribution, potentially altering immune surveillance and therapeutic landscapes.

## 1 Introduction

### 1.1 The role of MHC molecules in peptide presentation

The immunopeptidome is the assortment of peptides bound to MHC molecules presented on cell surfaces (1). The immunopeptidome dynamics include the accessibility of peptides across cellular vicinities, the turnover by a peptide-loading complex, and the intricacies of antigen processing and presentation (APP) components (2). Thus, the characteristics of peptides, such as their affinities for MHC molecules, half-lives, and dissociation rates, play a crucial role in determining the landscape of the immunopeptidome (3). Once peptides with sufficient affinity are bound to MHC molecules, they are transported to the cell surface for presentation over extended periods. Upon detachment, MHC molecules are typically internalized and degraded. While it has been suggested that MHC molecules may participate in the reloading and re-presentation of new peptides, this is believed to be a relatively minor phenomenon (4). Additionally, MHC molecules can be released from the cell surface as soluble entities or bound to EVs (5). This release presents the potential for MHC molecules to influence immune responses at remote locations within the body, thereby demonstrating the broad impact of this research (6).

### 1.2 Emerging insights: ncRNA-derived peptides and extracellular vesicles

ncRNA-derived peptides are a novel class of peptides originating from previously considered non-coding RNAs, including long non-coding RNAs (lncRNAs) and circular RNAs (circRNAs), which were initially believed to have no role in protein production. However, emerging evidence suggests that some ncRNAs possess internal ribosome entry sites (IRES) that facilitate their translation into small, functional peptides. MHC molecules can present these peptides, which are now considered part of the immunopeptidome and thus can influence immune recognition and response. This groundbreaking discovery challenges conventional views and opens new avenues for understanding immune responses. Importantly, not all ncRNA-derived peptides come from truly “non-coding” regions, as some may result from previously unannotated or cryptic open reading frames (ORFs) (7).

A recent analysis of MHC class I-bound immunopeptidome of human and mouse cancer cells identified a significant proportion of peptides encoded by lncRNA genes (8). T cells are crucial in monitoring the immunopeptidome, identifying ncRNA-derived peptides, and initiating immune responses. This process helps eliminate diseased cells and triggers B cell proliferation. While beneficial, T cell activation can also lead to immune suppression, tolerance, and pathological conditions in autoimmune and autoinflammatory disorders (9). Understanding their role in this context is essential for advancing immunology.

ncRNA-derived peptides diversify the antigenic landscape monitored by T cells, providing new targets for immune recognition. The discovery that a significant portion of the MHC-bound immunopeptidome is derived from ncRNAs suggests these peptides may play a previously underappreciated role in tumor immune evasion and immune system regulation. As researchers continue to investigate how these peptides interact with T cells, this area holds great promise for developing novel immunotherapies and diagnostic tools that can leverage the unique immunogenic properties of these peptides in a disease-specific context. This potential for novel immunotherapies and diagnostic tools should inspire hope and optimism in the scientific community.

The role of EVs is significant in transporting ncRNA-derived peptides and MHC molecules, spreading immune signals throughout the body, and contributing to immune surveillance. Research has focused on understanding the immunopeptidome, particularly the interaction between traditional peptides, ncRNA-derived peptides, and EVs, to identify pathogenic peptides, evaluate their immune potential, and explore their clinical applications.

**Figure 1:**
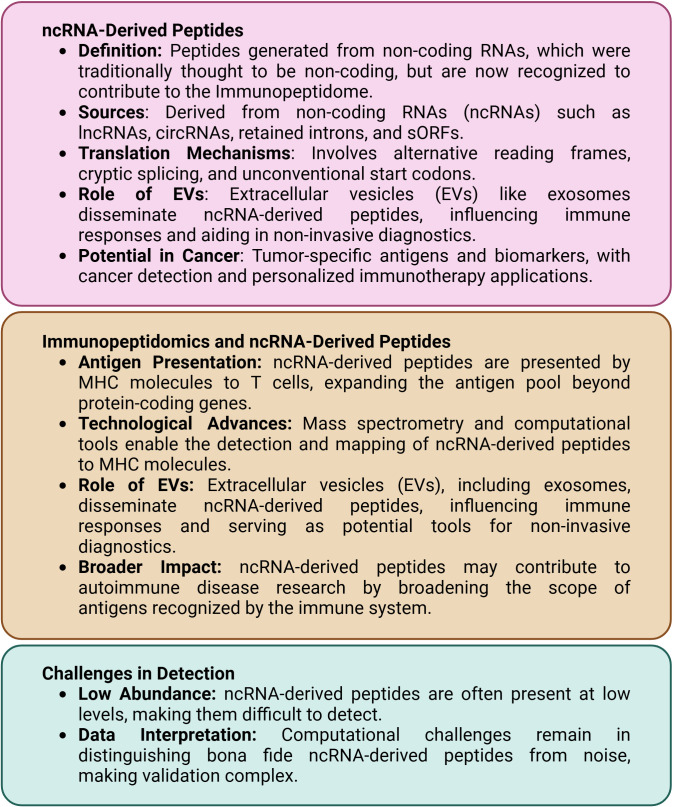
Key concepts in immunopeptidomics of ncRNA-derived peptides.

**Figure 1** provides an overview of critical concepts in immunopeptidomics, focusing on the role of ncRNA-derived peptides and their contributions to antigen presentation to help readers better understand the fundamental principles underlying these processes.

### 1.3 Diversity and polymorphism in MHC molecules

While HLA class I cancer antigens have traditionally been a significant focus in cancer immunotherapy, the role of class II molecules has been increasingly recognized as crucial in immunotherapeutic strategies. Studies reveal that certain MHC allotypes confer considerable resistance to pathogens, making it essential to understand the allotype-specific HLA peptidome, particularly concerning ncRNA-derived peptides, to uncover the genetic basis of this resistance in human and animal populations. This understanding of MHC diversity is not just important; it is urgent, as it can significantly impact our ability to combat diseases (10–12).

Genetic variants in MHC-associated genes, such as ERAP1, may predispose individuals to autoimmune diseases, yet these variants may persist in populations due to their protective effects during past epidemics (13–15). Given that ncRNA-derived peptides may contribute to autoimmune processes, understanding how different MHC allotypes present these noncanonical peptides is essential. Certain MHC allotypes may present ncRNA-derived peptides in ways that either trigger or dampen autoimmune responses. Additionally, specific HLA allotypes have been linked to disease resistance and susceptibility, emphasizing the need to understand the unique peptidomes and individual T cell receptor (TCR) repertoires associated with these allotypes (16).

### 1.4 Challenging traditional views: the composition of the immunopeptidome

Encompassing the role of ncRNAs in a deeper understanding of antigen processing and presentation is required for more granular prediction of presented peptides (17). Research into the biogenesis of the immunopeptidome—especially contributions from extracellular vesicles and ncRNA-derived peptides—offers significant implications for our understanding of immune responses (18).

The source proteome of the MHC peptidome includes a diverse array of cellular proteins originating from various organelles, such as the nucleus and mitochondria (19). The immunopeptidome is shaped not solely by the law of mass action but reflects complex pathways that integrate contributions from a range of proteins—mature, long-lived, short-lived, and incompletely synthesized (20). Some peptides are templated directly from DNA or RNA, while others result from post-translational modifications (PTMs). This dynamic interplay highlights the necessity of including ncRNA-derived peptides and extracellular vesicles in immunopeptidome research (21, 22).

Understanding how different peptide sources influence T-cell recognition and immune activation may lead to innovative strategies for enhancing vaccine efficacy and precision medicine approaches.

## 2 Advancements in immunopeptidome analysis

### 2.1 Understanding the composition and complexity of the immunopeptidome

The immunopeptidome is a vast and intricate collection of peptides presented by MHC molecules on cell surfaces, originating from various cellular proteins across different compartments (23). It may exhibit preferences for specific “hotspot” regions in the genome (24). This complexity includes peptides from traditional protein-coding genes and non-canonical sources, such as ncRNAs (25). Non-canonical peptides significantly enrich the immunopeptidome’s diversity, challenging established antigen-presentation paradigms.

Predicting the immunopeptidome’s composition, including peptide identities, relative abundance, and PTMs, is a formidable challenge. The potential peptide sequences that could bind to each MHC allele far exceed those presented, a complexity further heightened by non-canonical peptides that often exhibit distinct PTMs (26). Mass spectrometry (MS) has become essential for identifying and characterizing canonical and non-canonical peptides. The process typically starts with the immunoaffinity purification of MHC molecules, followed by peptide extraction. While techniques like Edman degradation were historically used for sequencing (27), liquid chromatography-tandem mass spectrometry (LC-MS/MS) is now the gold standard (28).

### 2.2 Mass spectrometry and AI in immunopeptidome research

Mass spectrometry is a powerful analytical technique used to measure the mass-to-charge ratio of ions, enabling the identification and quantification of molecules in complex mixtures. Since its inception, mass spectrometry has evolved significantly, with advancements in instrument design and ionization techniques leading to enhanced sensitivity and resolution. The 1990s marked a significant milestone in this journey when Hunt et al. (29) pioneered the sequencing of the immunopeptidome, revealing the mechanisms of peptide presentation by MHC molecules.

With the rise of personalized medicine, accurately identifying peptide neoantigens has become critical to ensure the effectiveness of therapy. The integration of LC-MS/MS with advanced high-resolution instruments, such as Orbitrap and time-of-flight (TOF) spectrometers, has significantly enhanced the sensitivity and accuracy of immunopeptidome studies (30, 31). These high-resolution technologies are crucial for distinguishing between peptides with similar masses and identifying low-abundance non-canonical peptides (32).

However, accurately identifying non-canonical peptides remains a primary challenge due to their limited re-presentation in standard protein databases (33, 34) and also because they are less abundant and may undergo unique PTMs, resulting in suboptimal fragmentation patterns during MS analysis (35).

AI and deep learning technologies have been increasingly integrated into MS data analysis to tackle these challenges. These AI-driven tools leverage large datasets of peptide spectra, enhancing the identification process by predicting non-canonical peptide sequences with greater accuracy (36–38). AI approaches also allow flexible management of false discovery rate (FDR) thresholds. While a strict FDR of 0.01 (1%) is standard, AI tools can confidently handle higher FDR thresholds without significantly increasing the risk of false positives. This capability makes them particularly advantageous for detecting rare non-canonical peptides that might otherwise be overlooked (39).

In this context, mass spectrometry is the foundational peptide identification technology. However, AI’s synergistic integration with mass spectrometry enhances the analysis by improving accuracy and flexibility, particularly in the challenging detection of non-canonical peptides. This integration is not just a technological advancement but a significant leap in AI in our understanding of the immunopeptidome, revealing a broader spectrum of peptides that MHC molecules can present.

### 2.3 Alternative methods for peptide recovery and analysis

In addition to traditional immunoaffinity purification, methods like mild acid elution (MAE) have been developed to recover MHC-bound peptides (40). MAE exposes cell surfaces to mildly acidic conditions, releasing peptides from MHC molecules without detaching the MHC heavy chains (41, 42). While MAE can yield large pools of peptides, including those associated with EVs, the quality and composition often differ from those obtained via immunoaffinity purification (23).

MAE-recovered immunopeptidomes may contain a significant fraction of non-ligand peptides, complicating analysis (43). However, this method is valuable for enhancing the detection of newly presented peptides after viral infections (44). Additionally, screening yeast or mammalian display libraries has been used to identify a broader range of MHC peptides (45). These libraries consist of cells transfected with DNA libraries encoding various MHC allotypes and peptide sequences, making them practical for discovering novel ncRNA-derived peptides that may influence immune responses (46). Notably, peptides arising from alternative splicing or cryptic translation start sites within ncRNAs represent a largely unexplored frontier in immunopeptidomics.

As previously discussed, identifying post-translational modified peptides through MS presents an ongoing challenge. Strategies such as enriching these peptides after immunoaffinity purification and incorporating potential PTMs as variables in sequence databases are utilized; however, these approaches do not fully address the complexities involved. Therefore, validating data for PTM MHC peptides, especially those derived from ncRNAs, remains a critical area of focus.

### 2.4 Identification and annotation of ncRNAs

The human translatome likely contains many undiscovered ORFs from various linear transcript translation frames further enriched by the thousands of novel unannotated ORFs (nuORFs) identified in the immunopeptidome. These ORFs include antigens derived from various transcripts, such as cancer germline, oncogenic missense mutation, frameshift mutation, splice site, gene fusion, and cancer-associated viruses (47, 48). Additionally, there are epitopes not encoded by the genome, known as noncanonical neoepitopes. These neoepitopes can arise from cellular processes specifically altered or induced in cancer, simultaneously resulting in a wide variety of neoantigens.

The mechanisms contributing to these noncanonical neoepitopes include alternative splicing, post-translational modification, RNA editing, and aberrant mRNA translation. This approach was recently reviewed (49, 50). These neoepitopes contrast sharply with classic neoantigens, genetically encoded and produced from only one mutated gene (mRNA or ncRNA) at a time.

Furthermore, circRNA-derived ORFs, which span the backsplice junction (BSJ), add another layer of non-genomic neoantigens that may also be classified as post-transcriptionally derived neoepitopes. This novel class of noncanonical neoepitopes could be particularly valuable in cancers characterized by a low mutational burden, which are thought to evade immune detection due to the limited availability of classic neoantigens (51).

Neoantigens arising from somatic mutations have become pivotal targets in personalized cancer therapies. MHC molecules present these novel protein fragments on cancer cell surfaces, distinguishing them from normal cells and making them prime candidates for immunotherapy. While traditional neoantigen research has focused on peptides derived from protein-coding regions, recent studies highlight the potential of ncRNAs to generate immunogenic peptides, expanding the antigen repertoire. This section explores methodologies for identifying, predicting, and validating these ncRNA-derived neoantigens, integrating computational tools with experimental approaches to harness their therapeutic potential.

The crux of ncRNA-derived neoantigen discovery lies in accurately identifying and annotating ncRNAs within the cancer genome, and it typically begins with high-throughput RNA sequencing (RNA-Seq), capturing the full spectrum of the transcriptome, including both coding and noncoding RNAs. Computational tools like CPC2 (Coding Potential Calculator 2), LncADeep, and FEELnc (Finding ncRNAs by Estimating their Likelihood) facilitate the classification of ncRNAs by analyzing sequence features, structural motifs, and the absence of long ORFs (52, 53).

In addition to identifying lncRNAs, these tools are essential for detecting circRNAs formed through back-splicing events. Tools like circRNA_finder and CIRI2 are crucial for annotating circRNAs by identifying back-splice junctions, a hallmark feature of these molecules (18). Updated resources like circAtlas 3.0 provide comprehensive annotations of circRNAs across multiple species, facilitating the identification of circRNA-derived neoantigens (18, 54).

To support ncRNA-derived immunopeptidome research, **Table 1** provides an overview of the critical databases and bioinformatics tools currently available for identifying and analyzing ncRNAs, aiding in discovering and characterizing non-canonical peptides.

**Table 1:** Key databases and tools for ncRNA-derived immunopeptidome research.

Name	Type	Focus	Biological Specimen	URL	Reference
IEDB	Database	Immunogenic peptides, including ncRNA-derived peptides	Various human and animal studies	https://www.iedb.org/	(55)
LNCipedia	Database	Annotated lncRNAs	Various tissues and cell lines	https://lncipedia.org/	(56)
circBASE	Database	circRNAs	Various tissues and cell lines	http://www.circbase.org/	(57)
SysteMHC Atlas v2.0	Atlas	System-wide MHC-associated peptide presentation	Multiple biological specimens	https://systemhc.sjtu.edu.cn/	(58)
FuncPEP v2.0	Tool/Database	Functional annotation of peptides	Human tissues	https://bioinformatics.mdanderson.org/Supplements/FuncPEP/about.html	(59)
ExoCarta	Database	Exosomal content, including proteins, lipids, and RNA (including ncRNAs)	Exosomes from cell lines, blood, urine, and other fluids	http://www.exocarta.org/	(60)
Vesiclepedia	Database	Extracellular vesicles, including proteins, lipids, and RNA content	Cell lines, bodily fluids, and tissues	http://www.microvesicles.org/	(61)
OpenProt	Database/Atlas	Non-canonical proteins and peptides, including ncRNA-derived peptides	Human and model organisms	https://www.openprot.org/	(62)
circAtlas 3.0	Atlas	circRNAs across multiple species	Multiple species	https://ngdc.cncb.ac.cn/circatlas/	(54)
SPENCER	Tool	Prediction and validation of small peptides encoded by sORFs	Various tissues and cell lines	https://spencer.renlab.org/#/home	(63)
ncEP	Tool	Prediction of non-canonical epitopes from ncRNAs	Various tissues and cell lines	http://www.jianglab.cn/ncEP/	(64)
CPC2	Tool	Coding potential calculator	Various tissues and cell lines	http://cpc2.cbi.pku.edu.cn/	(65)
RiboTaper	Tool	Analysis of ribosome profiling data	Various tissues and cell lines	https://ohlerlab.mdc-berlin.de/software/RiboTaper_126/	(66)
LncPep	Database	Small peptides encoded by lncRNAs	Various tissues and cell lines	http://www.shenglilabs.com/LncPep/	(67)
PRIDE	Repository	Comprehensive proteomics data, including ncRNA-derived peptides	Various biological specimens	https://www.ebi.ac.uk/pride/	(68)
miRandola	Database	Circulating microRNAs, including EV-associated RNAs and potentially derived peptides	Blood, serum, plasma, and other bodily fluids	http://mirandola.iit.cnr.it/	(69)
HuVarBase	Database	Human sequence variants, including ncRNA variants leading to non-canonical peptides	Various tissues and cell lines	https://www.iitm.ac.in/bioinfo/huvarbase/	(70)

Despite advancements in ncRNA identification and annotation, several challenges remain. The complexity of the transcriptome, along with the presence of overlapping genes and variable splicing patterns, can complicate the accurate classification of ncRNAs. Additionally, many ncRNAs have tissue-specific expression profiles, making it essential to analyze samples from various biological contexts to understand their roles comprehensively (71).

Furthermore, distinguishing functional ncRNAs from transcriptional noise or high throughput sequencing artifacts poses a significant challenge, as many ncRNAs exhibit low expression levels and may be produced only in specific cellular conditions (72). As the field progresses, continued efforts in refining computational methods and enhancing experimental techniques will be essential for unraveling the full spectrum of ncRNAs and their functional implications.

### 2.5 Prediction of ncRNA translation and HLA presentation

Following the identification of ncRNAs, the next step involves predicting their potential to be translated into immunogenic peptides. Ribosome profiling (Ribo-Seq) has become an essential technique, offering a snapshot of actively translated regions within the transcriptome. This technique has revealed that many ncRNAs, previously classified as noncoding, can produce functional peptides (50).

Computational tools like ORF-RATER and RiboTaper analyze Ribo-Seq data to identify sORFs within ncRNAs that may encode neoantigens (18, 26). These sORFs are then evaluated for HLA binding predictions using algorithms such as NetMHCpan and MHCflurry, which assess the likelihood of peptide binding to specific HLA alleles (73). This binding is crucial for peptides to be presented on the cell surface and recognized by the immune system.

Additionally, specialized tools like SPENCER and ncEP predict and validate small peptides encoded by sORFs within ncRNAs. SPENCER integrates ribosome profiling and mass spectrometry data to confirm peptide translation, while ncEP focuses on predicting non-canonical epitopes from ncRNAs that HLA molecules could present (74).

Experimental validation is essential to confirm the immunogenic potential of ncRNA-derived neoantigens predicted through computational methods. This process typically involves several steps. Predicted peptides are synthesized and tested for binding affinity to specific MHC molecules using assays like fluorescence polarization and enzyme-linked immunosorbent assay (ELISA). These assays help validate computational predictions. The immunogenicity of the peptides is assessed by testing their ability to activate T cells through assays such as T-cell proliferation or interferon-gamma (IFN-γ) release assays, providing direct measures of the peptide’s potential to elicit an immune response. Integrating mass spectrometry with proteogenomics allows for direct detection of ncRNA-derived peptides and their presentation by MHC molecules. Tools like MaxQuant and MSFragger analyze MS data, offering a high-throughput method for validating the physical presence of these peptides.

### 2.6 Integrative proteogenomic approaches

Proteogenomics integrates proteomic data with genomic and transcriptomic information, essential for validating ncRNA-derived neoantigens. This approach ensures that peptides predicted by computational tools are synthesized and presented on cell surfaces. ProteoMapper and OpenProt map predicted peptides to their genomic origins, confirming their existence (33).

Robust databases are vital for cataloging these novel antigens. The Immune Epitope Database (IEDB) offers a comprehensive repository of immunogenic peptides, including those from ncRNAs (55). At the same time, LNCipedia and circBase provide extensive collections of annotated lncRNAs and circRNAs, aiding in neoantigen identification (42, 44). SysteMHC Atlas v20 includes data on canonical and non-canonical peptides, and FuncPEP v20 predicts the functional impact of non-canonical peptides (75).

Not all predicted MHC-binding peptides are immunogenic, and experimental validation is resource-intensive. Nonetheless, the patient-specific nature of ncRNA-derived neoantigens makes them ideal for personalized cancer vaccines, enhancing immunotherapy efficacy (74). These neoantigens could also be combined with other immunotherapies to improve patient outcomes (50).

### 2.7 Future directions and significance of immunopeptidome research for immunotherapy

The integration of mass spectrometry and AI technologies in immunopeptidome research is revolutionizing the identification and characterization of ncRNA-derived neoantigens. By employing advanced techniques such as LC-MS/MS, Ribo-Seq, and integrative proteogenomics, researchers are uncovering a rich landscape of immunogenic peptides that can be harnessed for personalized cancer therapies. Targeted MS techniques, such as parallel reaction monitoring (PRM) and selected reaction monitoring (SRM), are also essential for accurately detecting low-abundance peptides (73). To ensure reliability, validating non-canonical peptides requires corroboration of MS findings with experimental methods.

Despite the challenges associated with identifying and validating these non-canonical peptides, ongoing advancements in bioinformatics tools and experimental methodologies are promising for enhancing immunotherapy strategies. The discovery and characterization of ncRNA-derived neoantigens could provide novel insights into immune responses and pave the way for innovative and effective therapeutic approaches.

## 3 Advancements in immunopeptidome composition and presentation

### 3.1 Immunopeptidomes’ origin and biomaterial

The immunopeptidome, encompassing peptides presented by MHC molecules, originates from diverse sources such as cultured cells, tissues, tumors, and biofluids. Among these sources, EVs have gained particular attention for their role in modulating immune responses by presenting ncRNA-derived peptides (6). EVs, including exosomes and microvesicles, function as vehicles for intercellular communication, carrying various molecular cargos such as proteins, lipids, and nucleic acids, including ncRNAs (6).

As one of the pivots in tumor immunology, EVs are crucial in presenting tumor-specific antigens, including neoantigens derived from ncRNAs. Research has identified these tumor-associated peptides in cultured cancer cells and tumor tissues, with valuable insights from patient-derived xenograft (PDX) models (61). For instance, studies have explored the potential of EVs in enabling non-invasive cancer diagnostics and monitoring treatment responses by detecting tumor-derived peptides.

Although many peptide sequences from these studies, including EV-associated peptides, have been deposited in public databases like the Human MHC Project and the Human Immunopeptidome Project, no repository is dedicated solely to ncRNA-derived immunopeptides from EVs. Existing databases like Vesiclepedia (61), ExoCarta (60), and LncPep (67) provide valuable data on the proteome, lipidome, and RNA cargo of EVs but do not comprehensively cover the landscape of ncRNA-derived immunopeptides associated with EVs.

The relationship between the immunopeptidome, proteome, and transcriptome is complex. Studies have shown poor correlations between the relative abundance of MHC peptides and their source proteins or degradation rates. However, slightly higher correlations are observed between source protein levels and the number of HLA peptides identified (76), suggesting that factors beyond simple protein degradation influence the immunopeptidome, such as protein synthesis rates, post-translational modifications, and the presence of ncRNA-derived peptides.

The theoretical implications of peptide diversity in these contexts have direct clinical outcomes. For example, the ability of EVs to transport ncRNA-derived peptides, including tumor-specific neoantigens, across various cellular environments opens new avenues for cancer diagnostics and therapies. Identifying such peptides in patient samples can serve as biomarkers for early cancer detection or therapeutic targets in precision medicine.

**Figure 2:**
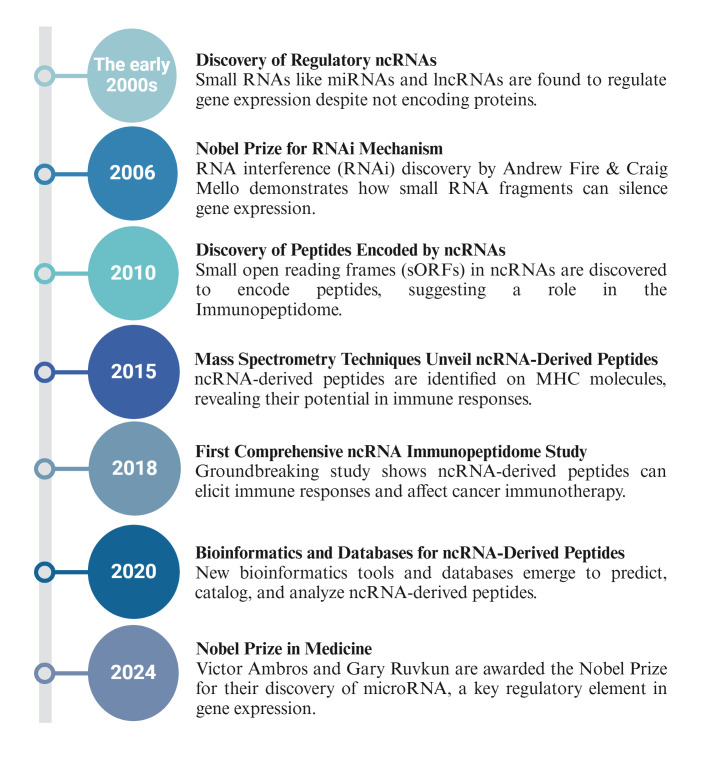
Historical timeline of ncRNA-derived immunopeptidome research.

**Figure 2** provides a historical timeline highlighting key milestones and discoveries in this growing field to contextualize the development of ncRNA-derived immunopeptidome research (22, 77–80).

### 3.2 Canonical and non-canonical immunopeptidomes

Non-canonical MHC peptides arise from sORFs, alternative reading frames, frameshifting, and retained intron sequences. They often come from sequences not annotated in the genome, contributing to the diversity of peptides presented by MHC molecules (81).

Ribosome profiling has provided strong evidence for the prevalence of non-canonical peptides derived from unannotated RNA sequences. This high-resolution technique reveals translation events from previously thought to be noncoding sequences, resulting in MHC-bound peptides recognized by T cells that elicit an immune response (82).

The clinical relevance of this peptide diversity is highlighted in cancer immunotherapy, where understanding the complex mechanisms of ncRNA-derived peptides has led to novel therapeutic approaches. Peptides derived from lncRNA HOXB-AS3, for instance, inhibit the oncogenic function of the HOXB gene in colon cancer, showing how non-canonical peptides can directly affect tumor progression and offer therapeutic targets (83).

### 3.3 The role of sORFs in ncRNA-derived neoantigens

**Figure 3:**
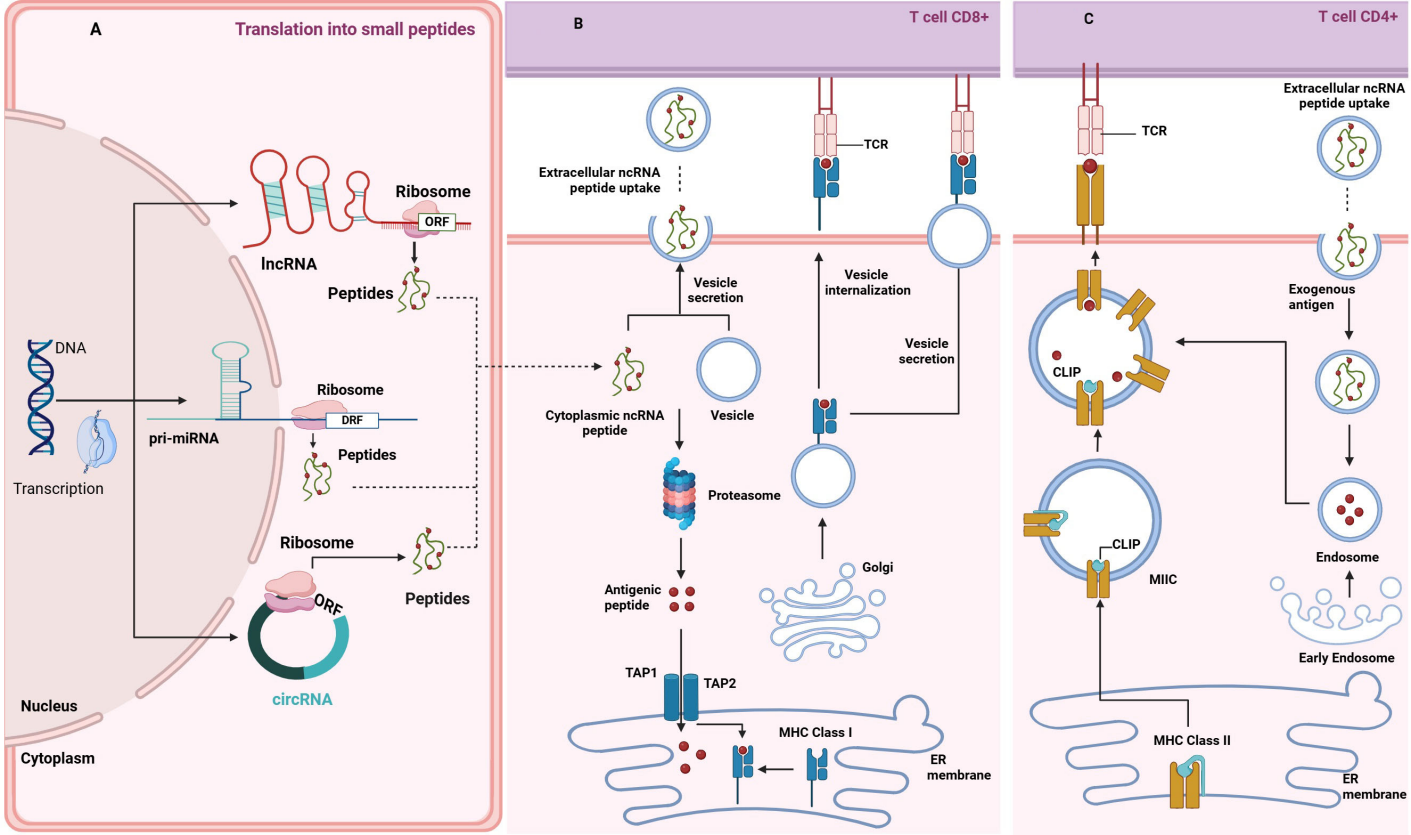
Crosstalk between ncRNA-derived peptides and antigen presentation mediated by extracellular vesicles. **(A)** Source of ncRNA-Derived Peptidome: The panel illustrates how peptides are derived from ncRNAs within different types of ncRNA transcripts, depicting the processing pathways through which ncRNAs are translated into functional peptides. The illustration highlights the cellular components of RNA processing, including microRNA maturation and peptide translation via ribosome complexes. **(B)** MHC I Intracellular Peptide Load: The panel illustrates the process of peptide loading and presentation by MHC Class I molecules to CD8+ T cells, describing the intracellular mechanisms involved, starting with either loading endogenous, ncRNA-derived polypeptides into EVs (see next panel), or their breakdown in the proteasome - an enzymatic complex in the cytosol, into shorter antigenic peptides (depicted as red dots). Those peptides are then transported to the ER by TAP1/2. The peptides are loaded onto newly synthesized MHC I molecules at this stage. The subsequent transport of these complexes through the Golgi apparatus to the cell surface for antigen presentation to CD8+ T cells via TCR binding is shown. Arrows indicate the direction of peptide transport and the pathways involved in presentation. **(C)** MHC II Extracellular Peptide Uptake: The panel shows the uptake and presentation of extracellular ncRNA peptides via MHC Class II molecules to CD4+ T-cells. Those peptides are internalized within extracellular vesicles and processed by the endosomal pathways into short antigenic peptides (depicted as red dots). MHC-II dimers are assembled in the ER and bound to the invariant chain (Ii), preventing premature peptide binding. The Ii-MHC-II complex is transported to the MHC-II compartment (MIIC), where endosomal proteases degrade Ii into class II-associated invariant chain peptide (CLIP), occupying the peptide-binding groove. ncRNA-derived antigenic peptides bind to MHC-II, replacing CLIP. The resulting peptide-MHC complexes are then transported to the cell surface, where they are recognized by CD4+ T cells.

Small open reading frames encode peptides shorter than 100 amino acids and can be found in lncRNAs and circRNAs (**Figure 3A**). The discovery that ncRNAs can harbor sORFs capable of producing immunologically relevant peptides has expanded our understanding of the noncoding genome’s role in cancer biology.

A particularly striking example is the circRNA circ0076651, hosted by the HSP90AB1 gene, detected exclusively in lung cancer specimens but absent in normal tissues (84), as it demonstrates the potential of circRNA-derived peptides in biomarker development for cancer diagnostics. Additionally, microRNAs (miRNAs), often known for gene regulation, can encode micro peptides that regulate immune responses. For instance, miPEP31, encoded by pri-microRNA-31, is highly expressed in Foxp3+ regulatory T cells (Tregs), where it promotes Treg differentiation (84), highlighting the role of ncRNA-derived peptides in immune regulation.

### 3.4 Mechanisms of non-canonical peptide production

The production of non-canonical peptides involves unique mechanisms such as cryptic translation, alternative splicing, and post-transcriptional modifications. Cryptic translation allows the synthesizing of peptides from sORFs that are not traditionally recognized as coding sequences. This translation often occurs without the canonical AUG start codon and can be initiated by non-AUG codons.

For example, cryptic antigenic peptides encoded by tumor-specific circRNA circFAM53B have shown a potential to induce antitumor immunity in breast cancer (85). Similarly, post-transcriptional modifications like adenosine-to-inosine (A-to-I) RNA editing can create novel sORFs, generating peptides with immunogenic properties (86).

### 3.5 Other sources of non-canonical immunopeptidome targets

Families of transposable elements (TEs) comprise a significant portion of the human genome, with estimates suggesting that they make up about 50% of the DNA, often dismissed as “junk DNA.” However, recent research has revealed that these TEs are now emerging as a potential source of unique peptides that could trigger immune responses against tumors. In particular, the reactivation of TEs has been observed in patients undergoing immunotherapies, where they produce novel tumor-specific antigens that are subsequently presented via human leukocyte antigen (HLA) molecules.

In addition to TEs, other genomic elements, such as pseudogenes - non-functional copies of genes, also generate non-canonical immunopeptidome targets. Pseudogenes can become aberrantly expressed under certain conditions, producing unconventional peptides that may serve as neoantigens, further broadening cancer immunotherapy targets (as detailed earlier).

This growing body of evidence underscores the underestimated potential of what has traditionally been considered the genome’s “dark matter.” These elements now have the potential to shed light on previously overlooked immunological targets. (87).

### 3.6 Challenges and opportunities in exploiting ncRNA-derived neoantigens

Identifying and exploiting ncRNA-derived neoantigens poses several challenges but also offers significant opportunities. Traditional methods for predicting neoantigens focus on mutations within protein-coding genes and are unsuited for detecting ncRNA-derived peptides. Therefore, new bioinformatics tools and experimental techniques are needed to predict and validate these non-canonical peptides accurately.

One promising approach is Ribo-Seq, which provides a snapshot of actively translated regions of the transcriptome, including ncRNAs. Coupling Ribo-Seq with mass spectrometry can help identify and characterize ncRNA-derived peptides presented by MHC molecules on the surface of tumor cells (88). Additionally, high-throughput screening techniques and machine learning algorithms are being developed to predict the coding potential of ncRNAs and identify sORFs that may serve as sources of neoantigens.

The clinical potential of this research is vast. Targeting ncRNA-derived peptides allows for more personalized and precise immunotherapies, enhancing treatment outcomes in various cancers, particularly in tumors with low mutational burden where traditional neoantigens are scarce (74). This peptide diversity expands the repertoire of potential therapeutic targets and provides new tools for diagnostics and disease monitoring, mainly through non-invasive methods like EV analysis. As research progresses, understanding sORF translation and non-canonical peptide production will be crucial in harnessing their full potential for innovative immunotherapy strategies.

### 3.7 Translational potential of ncRNA-derived peptidome in clinical oncology

Recent advances in neoantigen research have accelerated the development and approval of tumor immunotherapies, including cancer vaccines, adoptive cell therapy, and antibody-based treatments, particularly for solid tumors. Neoantigens are formed by tumor cells due to alterations like genomic mutations and abnormal RNA splicing; thus, they are recognized as non-self and elicit immune responses that bypass tolerance mechanisms (89).

ncRNA inclusion into identifying and predicting tumor-specific neoantigens has improved significantly due to enhancements in next-generation sequencing mass spectrometry and bioinformatics (58). In the advancing field of immunopeptidomics, innovative methodologies have improved our ability to identify and characterize peptides presented by MHC molecules on cell surfaces. High-resolution mass spectrometry now allows for precise detection of peptides, even at lower abundances. Improvements in LC-MS/MS enable deeper coverage of the immunopeptidome from limited samples. Additionally, advanced bioinformatics tools predict MHC binding affinities and enhance peptide identification accuracy. Integrating genomic and transcriptomic data provides insights into the origins and functions of these peptides in immune recognition. These advancements are essential for understanding tumor immune landscapes, guiding peptide-based vaccine design, and developing personalized immunotherapies that target specific cancer antigens, ultimately improving patient outcomes. These methodological advances were recently comprehensively reviewed (74, 90–93).

Unlike tumor-associated antigens, neoantigens are highly immunogenic and specific to tumors, making them valuable targets for personalized cancer therapies and indicators of survival prognosis and responses to immune checkpoint blockade. Understanding the mechanisms behind neoantigen-induced immune responses will enhance cancer therapy development and implement neoantigen-based treatments (74).

Studies have shown that small open reading frames exist in ncRNA genes with peptide encoding potential (8, 83, 85, 94). Thus, the simultaneous deregulation of ncRNAome as a template for anti-cancer immunopeptide synthesis can be deployed as a novel anti-cancer vaccine. Studies also reassess the coding vs. non-coding potential of the genome for developing cancer immunotherapies (95–97). Several clinical and preclinical models have identified neoantigens unique to tumor cells as primary sources of tumor-specific antigens (TSAs) that activate anti-tumor immunity through cytotoxic T lymphocytes (CTLs) (8, 98–101). Identifying these antigenic peptides is essential for developing anti-tumor therapies, such as vaccines and engineered T-cell treatments (85). While earlier research focused on nonsynonymous mutations in the protein-coding genome to find TSAs, many cancers have low mutational burdens. Therefore, it is crucial to explore other methods for identifying tumor-specific peptides. One promising approach is using peptides encoded by ncRNAs, which are abundant in malignant cells and distinct from those found in normal tissues (85). These ncRNA-encoded peptides may stimulate antigen-specific T cells (85). However, the immunosuppressive tumor microenvironment can limit antigen presentation and T-cell activation.

Therapeutic vaccines utilizing ncRNA antigenic peptides or circRNAs may help enhance these processes. Researchers have identified HLA-I-binding antigenic peptides from tumor-specific circRNAs, circFAM53B, which effectively primed naive CD4+ and CD8+ T cells and induced anti-tumor immunity. The expression of circFAM53B and its peptides were linked to significant infiltration of CD8+ T cells and improved survival in breast cancer and melanoma patients (58). This study emphasizes the potential of tumor-specific ncRNAs for developing cancer vaccines, though further research is needed to assess their effectiveness as standalone or combined treatments (102, 103).

Certain ncRNAs can suppress tumor suppressor genes or modulate immune checkpoint pathways, which may facilitate tumor immune evasion. In contrast, peptides derived from ncRNAs—often processed into novel cancer-specific antigens—have been shown to activate T-cell responses, leading to tumor rejection in preclinical models of melanoma and lung cancer. These peptides represent potential targets for vaccine development. This is supported by preclinical research in which synthetic long peptides derived from tumor antigens have elicited strong immune responses in melanoma patients (104). Identifying such peptides is just the first step in creating a vaccine. The process becomes more complicated when combining MHC-decorated immunopeptidomes with adjuvants and delivery vehicles. Therefore, exploring naturally formed MHC complexes on the surface of EVs that can serve as vehicles while carrying adjuvants (e.g., PD-L1; (105)) may provide a valuable tool for delivering bioactive molecules that support antitumor immunity by presenting tumor antigens to immune cells. For example, dendritic cells loaded with tumor-derived exosomes have been used in clinical trials to stimulate immune responses in patients with advanced non-small cell lung cancer, demonstrating a novel therapeutic approach that leverages the natural antigen-presenting properties of these vesicles (106–108).

## 4 Biogenesis of the immunopeptidome

### 4.1 Biosynthesis of the immunopeptidome from non-templated sequences

As previously discussed, ncRNAs play a critical role in expanding the repertoire of potential neoantigens. In addition to sORFs, the emergence of spliced peptides is another intriguing aspect of the immunopeptidome. The clinical potential of this research is vast. Targeting ncRNA-derived peptides allows for more precise immunotherapies, enhancing treatment outcomes in various cancers, particularly in tumors with low mutational burden where traditional neoantigens are scarce (74). Although the prevalence of spliced peptides remains debatable, their potential contribution to the immunopeptidome is significant, particularly in the context of cancer, where the complexity of the tumor microenvironment demands diverse antigenic profiles.

In parallel, non-canonical peptides derived from various ncRNAs are also produced through non-canonical translation mechanisms (109). For example, when tryptophan levels are depleted due to elevated enzyme indoleamine 2,3-dioxygenase (IDO1) activity, the ribosome may misincorporate phenylalanine instead of tryptophan during translation. This misincorporation results in non-templated peptides, which significantly enhance the diversity of the immunopeptidome, particularly during immune responses. Together, the roles of spliced and non-canonical peptides highlight the dynamic nature of the immunopeptidome, underscoring its importance in generating a robust immune response against tumors (110).

Furthermore, as highlighted earlier, the role of EVs in disseminating ncRNA-derived peptides cannot be overstated, as they are crucial for transporting MHC-bound peptides that influence local and systemic immune responses (111). Their ability to transfer tumor-associated antigens derived from ncRNAs not only aids in immune surveillance but may also facilitate immune evasion, emphasizing their significance in tumor biology (112, 113).

By integrating the concepts of ncRNAs, spliced peptides, and EVs, researchers can gain deeper insights into the intricacies of the immune landscape.

### 4.2 ncRNA-derived peptides and proteasomal proteolysis

Traditionally, the generation of the immunopeptidome has been attributed to proteasomal proteolysis, which degrades the cellular proteome to produce peptides for MHC presentation (114). However, ncRNA-derived peptides challenge the notion that the proteasome is the sole source of MHC-bound peptides. The degradation of these ncRNA-derived peptides involves both the proteasome and other proteolytic systems, underscoring the complexity of their biogenesis (109, 115) (**Figure 3B**).

While the proteasome has been viewed as the primary source of peptides for MHC class I molecules, evidence shows that proteasome inhibition does not uniformly reduce MHC peptide presentation. In some cases, the presentation of specific peptides increases, suggesting the existence of alternative proteolytic pathways, such as those involving autophagosomes or lysosomal systems (116). Additionally, ncRNAs can modulate protein stability and degradation, further complicating the relationship between the proteasome and MHC peptide generation (117).

### 4.3 The role of EVs in immunopeptidome formation

Extracellular vesicles, including exosomes and microvesicles, play a pivotal role in the transport and presentation of ncRNA-derived peptides as evidenced by Transporter Associated with Antigen Processing (TAP) proteins, which are a class of transporter proteins present in EVs (15). By transporting MHC molecules loaded with ncRNA-derived peptides, EVs can influence immune surveillance and modulate responses in distant tissues. It is especially pertinent in cancer, where EVs may carry tumor-specific antigens that enhance anti-tumor immunity or help evade immune detection (50, 118).

Analyzing EVs from biofluids, such as blood or pleural effusions, provides insights into the systemic spread of ncRNA-derived peptides and their role in disease progression (119). This non-invasive approach not only aids in identifying potential biomarkers for early diagnosis but also opens new avenues for therapeutic interventions targeting the immunopeptidome.

### 4.4 Challenging the proteasome-centric dogma

Observations that proteasome inhibition does not uniformly reduce MHC peptide presentation indicate the existence of alternative pathways, such as those involving autophagosomes and specialized vesicular compartments, in generating the immunopeptidome (113).

The involvement of EVs in presenting ncRNA-derived peptides further complicates the conventional understanding of MHC peptide presentation (120). Delivering these peptides to antigen-presenting cells (APCs) and facilitating cross-presentation EVs alter the immune responses. This complexity highlights the need for a nuanced understanding of the sources and pathways involved in MHC peptide presentation.

The biogenesis of the immunopeptidome is a multifaceted process involving proteasomal proteolysis, alternative proteolytic pathways, and the critical role of EVs. ncRNA-derived peptides, presented through these diverse mechanisms, significantly contribute to the immune landscape, offering new insights into immune surveillance and evasion. As our understanding of these processes evolves, targeting ncRNA-derived peptides and EVs may become increasingly important in therapeutic interventions, particularly cancer treatment.

### 4.5 ncRNA-derived peptides and EVs in the conventional pathways of antigen presentation

Protein-coding gene-derived peptides (the peptidome) arise from proteins processed by the proteasome pathway - the primary enzyme responsible for degrading cellular proteins and generating peptide substrates in the cytosol. While short ncRNA-derived peptides may bypass some of the proteasome-mediated steps, it has been shown that treatment of melanoma cells with IFNγ or the proteasome inhibitor did not significantly alter the presentation of circRNA-BSJ-derived peptides in comparison to canonical peptides, suggesting they follow similar routes of antigen processing and presentation (94).

The proteasome thus downgrades proteins into peptide substrates for transport into the endoplasmic reticulum (ER), where these peptides are further trimmed by ERAP1/2 (ERAAP) before being loaded onto MHC class I molecules. Studies show that inhibiting the proteasome reduces MHC presentation, indicating a dependence on the proteasome for peptide availability (121, 122). However, recent findings suggest that the rate of expression of peptide-receptive HLA molecules may be the limiting factor in HLA presentation (123, 124). Additionally, immunoproteasome subunits are located within the MHC class II locus and are upregulated during inflammation, supporting the role of immunoproteasomes in producing suitable peptides for MHC presentation (125). Questions remain about the proteasome being the sole source of MHC ligands, as inhibitor studies reveal complex changes in the presentation of peptides derived from coding transcripts and ncRNA (126), suggesting that alternative pathways also contribute to peptide generation. The coordination between proteasome and lysosomal pathways and EV that also present MHC complex and TAP proteins is essential for cross-presentation, highlighting the complexity of this process. While the proteasome is vital in the immunopeptidome, other proteolytic systems in compartments like the autophagosome may also significantly influence various antigen-processing pathways (127, 128).

While it’s well-established that MHC class II molecules present peptides from extracellular proteins absorbed through phagocytosis, the claim that all such peptides originate externally is unsubstantiated (129, 130). Research shows these molecules also present peptides derived predominantly from intracellular proteins across various cell types. The exact contribution of intracellular versus extracellular sources to the MHC class II peptidome remains unclear and warrants further investigation. Notably, both MHC class I and II peptidomes often contain peptides from cells or tissues other than those presenting them, notably from dying cells, which are significant for cross-presentation (131). Finally, vesicular systems, including EVs and exosomes (132) but also processes like trogocytosis (133), and the transfer of peptides via gap junctions (134), also contribute to this dynamic.

## 5 The impact of therapeutic stress on immunopeptide presentation

### 5.1 Mechanisms of therapeutic stress

Immunotherapies, such as immune checkpoint inhibitors, adoptive T-cell therapies, and cancer vaccines, induce significant cellular stress, modifying the immune response and altering the immunopeptidome. These therapies can lead to oxidative stress, metabolic changes, and proteotoxic stress within tumor cells, impacting protein synthesis, degradation, and the repertoire of peptides presented by MHC molecules (135).

For example, immune checkpoint inhibitors, like anti-PD-1 and anti-CTLA-4 antibodies, block inhibitory signals on T cells, resulting in enhanced immune activation (136). Such heightened immune response fosters an inflammatory tumor microenvironment, which can increase proteolytic activity and subsequently change the immunopeptidome (137). Similarly, adoptive T-cell therapies promote targeted killing of cancer cells, inducing stress and antigen release. Stress from immunotherapy can upregulate proteasomal activity, leading to increased degradation of cellular proteins, including those from ncRNAs (138).

The enhanced proteolysis may expose cryptic peptides, typically hidden within larger protein structures, thereby increasing their availability for MHC binding and presentation. Therapeutic stress can also disrupt normal cellular processes, resulting in changes in PTMs such as phosphorylation, ubiquitination, and glycosylation (139). For instance, stress-induced phosphorylation of a protein can generate new epitopes recognized by the immune system (140).

### 5.2 The contribution of DRiPs to the immunopeptidome

Under stress, the rate of protein synthesis can be compromised, leading to the production of Defective Ribosome Products (DRiPs). These include peptides derived from ncRNAs that are quickly degraded and presented by MHC molecules (135). Increasing DRiPs can yield more diverse peptides, potentially enhancing the immune system’s ability to recognize tumor cells.

DRiPs, comprising MHC peptides from rapidly degraded cellular proteins, may represent a significant portion of the immunopeptidome. While estimates suggest that 30–70% of the immunopeptidome consists of DRiPs, methodological limitations make it challenging to determine their precise proportion (1). Identifying DRiPs-derived MHC peptides can be approached using dynamic-SILAC analysis, which tracks the incorporation and degradation of stable isotope-labeled peptides, helping to identify rapidly turned-over peptides indicative of their origin as DRiPs (141).

Furthermore, hypotheses suggest that specialized ribosomes may preferentially synthesize fragments of defective proteins for rapid degradation and MHC presentation. These specialized ribosomes have been referred to as “Immunoribosomes,” a subset of ribosomes predominately focused on generating proteins more efficiently targeted to antigen processing (Jonathan W. 142, 143). Compartmentalized production of peptides may also facilitate the effective management of unstable proteins, ensuring proper processing into immunogenic peptides (144). This strategy enhances the efficiency of antigen processing and cross-presentation, which is critical for initiating immune responses against intracellular pathogens or abnormal proteins produced by cancer cells. Despite extensive studies on EV-revised MHC-I peptides, a significant gap remains in demonstrating the role of extracellular vesicles in presenting DRiPs-derived peptides. This lack of evidence may stem from the complex biological mechanisms involved, experimental limitations, and a prevailing focus on other antigen-processing pathways that overshadow the potential contributions of EVs.

### 5.3 The impact of proteasome inhibition on the immunopeptidome

Therapeutic stressors, such as proteasome inhibition, can profoundly influence the immunopeptidome’s composition. Inhibiting the proteasome leads to cellular stress characterized by protein accumulation and depletion of free ubiquitin, which is crucial for MHC trafficking and antigen presentation (145).

This inhibition alters the repertoire of peptides presented by MHC molecules, with some peptides decreasing while others increase, indicating stress-induced changes in protein degradation pathways (146). Dying cells under stress do not effectively present MHC peptides, complicating the relationship between proteasomal activity and antigen presentation (147). Stress factors are crucial for cross-presentation, where APCs must degrade extracellular proteins in lysosomes and present them on MHC molecules (148). However, the complex proteolytic pathways suggest that matching cross-presented peptides with those produced endogenously is not guaranteed, especially under stress (149).

### 5.4 Examples of ncRNA-derived peptides under therapeutic stress

NcRNA-derived peptides, which arise from noncoding genome regions, are susceptible to changes in immunopeptide presentation under therapeutic stress. These peptides may be produced from alternative reading frames, sORFs, or other non-canonical translation events. Immunotherapy-induced stress can alter the expression and processing of ncRNAs, leading to the generation of novel peptides not typically present under normal conditions (150).

For instance, stress-induced modifications in the expression or processing of lncRNAs can lead to the presentation of unique peptides recognized by the immune system. Similarly, therapeutic stress can affect circRNAs, generating circRNA-derived peptides that contribute to the immunopeptidome (151). Cancer patients undergoing immune checkpoint blockade often show increased presentation of peptides derived from ncRNAs due to stress-induced changes in the tumor microenvironment (152).

Therapeutic strategies like oncolytic virus therapy can directly lyse cancer cells while modulating immune responses to the tumor microenvironment (153). This dual action offers a unique opportunity to evaluate ncRNA-derived peptides as targets for immune recognition. Under stress from therapies such as chemotherapy or radiation, the translation of ncRNAs into bioactive peptides is significantly altered, influencing cellular responses to therapy-induced damage (18, 83). These findings indicate that therapeutic stress can modulate the spectrum of ncRNA-derived peptides, unveiling novel targets for immunotherapy and enhancing anti-tumor responses.

### 5.5 Role of extracellular vesicles in immunopeptide presentation under stress

Immunotherapy-induced stress can significantly alter EV content and function, impacting their roles in immune surveillance and tumor antigen dissemination (154). Therapeutic stress may enhance EV release from tumor cells, increasing the dissemination of ncRNA-derived peptides. These EVs can interact with immune cells, such as dendritic and T cells, facilitating the transfer of stress-induced immunopeptides and promoting immune recognition. The ability of EVs to present stress-induced ncRNA-derived peptides highlights their potential as biomarkers for monitoring immunotherapy efficacy and as vehicles for therapeutic peptide delivery (155).

Moreover, EVs can transmit stress signals within the tumor microenvironment. By transferring stress-induced ncRNA-derived peptides to neighboring cells, EVs can amplify the immune response, enhancing overall immunotherapy effectiveness (155). This intercellular communication demonstrates EVs’ importance in therapeutic stress contexts and their potential to overcome tumor immune evasion.

### 5.6 Opportunities for novel immunotherapeutic strategies

Therapeutic stress significantly alters the immunopeptidome, particularly for ncRNA-derived peptides. This alteration can arise from various chemical, physical, and biological stresses, each uniquely influencing the mechanisms of immunopeptide presentation. Increased proteasomal activity, altered PTMs, and the production of DRiPs under stress highlight new therapeutic opportunities, particularly as ncRNA-derived peptides, typically minor components, become prominent targets for immune recognition.

The pivotal role of EVs in facilitating the transfer of ncRNA-derived peptides under stress enhances the immune system’s capacity to recognize and respond to cancer cells. This function opens avenues for innovative immunotherapeutic strategies, including developing vaccines targeting stress-induced neoantigens. Non-invasive tracking of ncRNA-derived peptides from EVs can yield real-time insights into tumor dynamics and therapeutic impacts.

Research into ncRNA-derived peptides under therapeutic stress enhances our understanding of cancer immunotherapy and emphasizes the need for advanced detection and characterization technologies. Integrating mass spectrometry with AI and machine learning can help overcome current limitations. Establishing a centralized database for ncRNA-derived peptides would facilitate research collaboration and accessibility.

While ncRNA-derived peptides present challenges, they also offer significant opportunities for advancing cancer immunotherapy. Addressing research gaps could improve our capacity to target these novel antigens, potentially leading to more effective and personalized treatments.

## 6 Contribution of noncanonical RNA-derived peptides and extracellular vesicles in MHC class I and II presentation

### 6.1 Noncanonical pathways of MHC class II peptide generation

Recent research has challenged the conventional view that all MHC class II peptides are derived from proteins taken up from the extracellular environment, particularly in the context of noncanonical RNA-derived peptides and their presentation through EVs. It is now understood that in cells expressing class II molecules, a significant proportion of the presented peptides originate from the degradation of intracellular proteins, including those encoded by ncRNAs (115). These ncRNA-derived peptides contribute substantially to the immunopeptidome.

### 6.2 The role of EVs in antigen transport and MHC peptide presentation

MHC class I and II peptidomes can include peptides from various cells or tissues, especially during cross-presentation, where antigens from dying cells provide rich sources (156). It has already been established that extracellular vesicles are pivotal in transporting ncRNA-derived peptides and MHC molecules between cells. They can carry fully formed MHC complexes loaded with these peptides, facilitating a process known as trogocytosis (157). **Figure 3C** shows that these EVs are crucial in antigen transport and presentation dynamics. Additionally, apoptotic vesicles containing partially degraded proteins can be processed by APCs for MHC presentation (130).

The balance between extracellular peptide uptake via EVs and the presentation of intracellularly expressed ncRNA-derived peptides is still under investigation. However, the role of EVs in delivering these peptides may redefine our understanding of immune responses, particularly in cancer immunotherapy, where identifying tumor-specific ncRNA-derived peptides presented by EVs could uncover novel therapeutic targets (118).

### 6.3 Potential for novel therapeutic approaches

Advancements in analytical techniques, such as dynamic stable isotope labeling and mass spectrometry, enable precise characterization of the immunopeptidome. These methods provide insights into how stress factors, including proteasome inhibition, influence peptide production and presentation dynamics (158). It is becoming clear that stress-induced changes in ncRNA translation and EV biogenesis are critical in shaping the peptide repertoire presented by MHC molecules (159).

Much of the previous research has utilized cultured cell lines, which may exhibit defects in antigen-processing pathways due to their cancerous nature (74), thus highlighting the need for studies in more physiologically relevant models to fully understand the immunopeptidome in normal and diseased tissues. As research progresses, insights gained from studying ncRNA-derived peptides and EV-associated antigens will be crucial for developing personalized immunotherapies and more effective vaccines against viral and other infectious diseases. Understanding the interplay between ncRNA translation, EV-mediated transport, and MHC presentation will pave the way for innovative therapeutic approaches in cancer and autoimmune diseases.

## 7 Conclusion

Therapeutic stress, particularly from immunotherapy, fundamentally transforms the immunopeptidome by altering the presentation of peptides, especially those derived from ncRNAs. These changes occur through various mechanisms, including enhanced proteasomal activity, altered post-translational modifications, and the production of defective ribosome products. As a result, ncRNA-derived peptides become more prominent under stress, presenting novel targets for immune recognition (13, 15–18).

PTMs tremendously increase the complexity of the proteasome, and glycosylation plays a significant role among them, impacting over 50% of mammalian proteins. Aberrations in protein glycosylation have been linked to many neurodegenerative disorders; thus, a deeper exploration into protein glycosylation can illuminate intricate molecular mechanisms that precipitate these conditions. For glycosylation, the leading technique up till today is LC-MS/MS. However, the current method of sequence-based searching falls short of glycan structure-based determination because of its limited occurrence. Spectral searching methods can utilize fragment intensity information, but difficulties hinder them in spectral libraries. Many new hybrid deep learning frameworks, such as DeepGP, can aid with this shortcoming. Still, a small database availability limits the deep learning model due to the lack of a standardized LC-MS/MS technique. This model only has information related to N-glycoproteins because of the even smaller available data for O-glycoproteins (160).

Extracellular vesicles play a crucial role in this process, facilitating the transfer of ncRNA-derived peptides and modulating the immune response under therapeutic stress (155, 156). Their potential as biomarkers and delivery vehicles underlines their significant impact on developing innovative immunotherapeutic strategies.

The dynamic nature of antigen presentation during therapeutic interventions, such as oncolytic virus therapy, diversifies the peptide landscape on MHC molecules, including peptides derived from ncRNAs generated through stress-related reprogramming of cellular translation and RNA processing (18, 159). These peptides, including potential neoantigens, could enhance the efficacy of immunotherapies by providing additional targets for T-cell recognition.

However, the characterization and identification of these novel antigens pose challenges due to the absence of a comprehensive database for ncRNA-derived peptides, similar to UniProt for canonical peptides. Deep data-independent acquisition (DIA) techniques, enhanced by AI tools like Prosit, play a vital role in overcoming these challenges. Deep DIA significantly enhances peptide discovery by enabling accurate peptide spectra and retention times predictions without relying solely on experimental libraries. This integration of in silico and experimental approaches broadens the scope of proteomics, yet the annotation of non-canonical peptides remains a pivotal hurdle to address (161).

AI innovations, exemplified by AlphaFold’s Nobel Prize-winning achievements, highlight transformative potential in addressing these limitations (162). Deep DIA and advanced AI-driven structural and functional analyses can refine identifying and characterizing ncRNA-derived peptides, supporting new avenues for biological insight. Advanced mass spectrometry is essential for tackling these challenges when paired with AI and machine learning. Together, these advancements promise to resolve barriers in this challenging research area, accelerating progress in proteomics and unlocking the potential of previously uncharted peptidomes. (31, 37, 38, 58, 161, 163). In conclusion, while ncRNA-derived peptides present unique challenges, they also offer significant opportunities for advancing cancer immunotherapy. Improving our detection capabilities and developing a centralized database for ncRNA peptides could significantly enhance our ability to target these novel antigens, leading to more effective and personalized therapeutic strategies. This research underscores the novel contributions of ncRNA-derived peptides and EVs to the immunopeptidome, paving the way for future studies and clinical applications.

## References

[1] Yewdell JW. MHC class I immunopeptidome: past, present, and future. Mol Cell Proteomics: MCP. (2022) 21:100230. doi: 10.1016/j.mcpro.2022.100230 35395404 PMC9243166

[2] Blum JS Wearsch PA Cresswell P. Pathways of antigen processing. Annu Rev Immunol. (2013) 31:443–73. doi: 10.1146/annurev-immunol-032712-095910 PMC4026165 23298205

[3] Micheletti F Bazzaro M Canella A Marastoni M Traniello S Gavioli R. The lifespan of major histocompatibility complex class I/peptide complexes determines the efficiency of cytotoxic T-lymphocyte responses. Immunology. (1999) 96:411–15. doi: 10.1046/j.1365-2567.1999.00707.x PMC2326763 10233722

[4] Hewitt EW. The MHC class I antigen presentation pathway: strategies for viral immune evasion. Immunology. (2003) 110:163–69. doi: 10.1046/j.1365-2567.2003.01738.x PMC1783040 14511229

[5] Maacha S Bhat AA Jimenez L Raza A Haris M Uddin S. Extracellular vesicles-mediated intercellular communication: roles in the tumor microenvironment and anti-cancer drug resistance. Mol Cancer. (2019) 18:555. doi: 10.1186/s12943-019-0965-7 PMC6441157 30925923

[6] Aloi N Drago G Ruggieri S Cibella F Colombo P Longo V. Extracellular vesicles and immunity: at the crossroads of cell communication. Int J Mol Sci. (2024) 25:12055. doi: 10.3390/ijms25021205 38256278 PMC10816988

[7] Jackson R Kroehling L Khitun A Bailis W Jarret A York AG. The translation of non-canonical open reading frames controls mucosal immunity. Nature. (2018) 564:434–38. doi: 10.1038/s41586-018-0794-7 PMC6939389 30542152

[8] Barczak W Carr SM Liu G Munro S Nicastri A Lee LN. Long non-coding RNA-derived peptides are immunogenic and drive a potent anti-tumour response. Nat Commun. (2023) 14:1078. doi: 10.1038/s41467-023-36826-0 36841868 PMC9968330

[9] Waldman AD Fritz JM Lenardo MJ. A guide to cancer immunotherapy: from T cell basic science to clinical practice. Nat Rev Immunol. (2020) 20:651–85. doi: 10.1038/s41577-020-0306-5 PMC7238960 32433532

[10] Evnouchidou I van Endert P. Peptide trimming by endoplasmic reticulum aminopeptidases: role of MHC class I binding and ERAP dimerization. Hum Immunol. (2019) 80:290–955. doi: 10.1016/j.humimm.2019.01.003 30682405

[11] Bassani-Sternberg M Chong C Guillaume P Solleder M Pak H Gannon PO. Deciphering HLA-I motifs across HLA peptidomes improves neo-antigen predictions and identifies allostery regulating HLA specificity. PloS Comput Biol. (2017) 13:e10057255. doi: 10.1371/journal.pcbi.1005725 PMC5584980 28832583

[12] Pierre P Laurent A-R Jung-Hua Y Julien P. Self-peptidome variation shapes individual immune responses - pubMed. Trends Genet. (2021) 37:414–20. doi: 10.1016/j.tig.2020.10.001 PMC7577255 33867017

[13] Scull KE Pandey K Ramarathinam SH Purcell AW. Immunopeptidogenomics: harnessing RNA-seq to illuminate the dark immunopeptidome. Mol Cell Proteomics: MCP. (2021) 20:100143. doi: 10.1016/j.mcpro.2021.100143 34509645 PMC8724885

[14] Raja A Kuiper JJW. Evolutionary immuno-genetics of endoplasmic reticulum aminopeptidase II (ERAP2). Genes Immun. (2023) 24:295–3025. doi: 10.1038/s41435-023-00225-8 37925533 PMC10721543

[15] Boyne C Lennox D Beech O Powis SJ Kumar P. What is the role of HLA-I on cancer derived extracellular vesicles? Defining the challenges in characterisation and potential uses of this ligandome. Int J Mol Sci. (2021) 22:135545. doi: 10.3390/ijms222413554 PMC8703738 34948350

[16] Joyce S Ternette N. Know thy immune self and non-self: proteomics informs on the expanse of self and non-self, and how and where they arise. Proteomics. (2021) 21:e2000143. doi: 10.1002/pmic.202000143 34310018 PMC8865197

[17] Trentini DB Pecoraro M Tiwary S Cox J Mann M Hipp MS. Role for ribosome-associated quality control in sampling proteins for MHC class I-mediated antigen presentation. Proc Natl Acad Sci United States America. (2020) 117:4099–41085. doi: 10.1073/pnas.1914401117 PMC7049129 32047030

[18] Wu P Mo Y Peng M Tang T Zhong Y Deng X. Emerging Role of Tumor-Related Functional Peptides Encoded by lncRNA and circRNA. Mol Cancer. (2020) 19:22. doi: 10.1186/s12943-020-1147-3 32019587 PMC6998289

[19] Synowsky SA Shirran SL Cooke FGM Antoniou AN Botting CH Powis SJ. The major histocompatibility complex class I immunopeptidome of extracellular vesicles. J Biol Chem. (2017) 292:17084–925. doi: 10.1074/jbc.M117.805895 PMC5641862 28860189

[20] Amoretti M Amsler C Bonomi G Bouchta A Bowe P Carraro C. Production and detection of cold antihydrogen atoms. Nature. (2002) 419:456–59. doi: 10.1038/nature01096 12368849

[21] Pearson H Daouda T Granados DP Durette C Bonneil E Courcelles M. MHC class I-associated peptides derive from selective regions of the human genome. J Clin Invest. (2016) 126:4690–701. doi: 10.1172/JCI88590 PMC5127664 27841757

[22] Wen K Chen X Gu J Chen Z Wang Z. [amp]]ldquo;Beyond traditional translation: ncRNA derived peptides as modulators of tumor behaviors. J Biomed Sci. (2024) 31:635. doi: 10.1186/s12929-024-01047-0 PMC11177406 38877495

[23] Mangalaparthi KK Madugundu AK Ryan ZC Garapati K Peterson JA Dey G. Digging deeper into the immunopeptidome: characterization of post-translationally modified peptides presented by MHC I. J Proteins Proteomics. (2021) 12:151–605. doi: 10.1007/s42485-021-00066-x PMC9807509 36619276

[24] Phulphagar KM Ctortecka C Jacome ASV Klaeger S Verzani EK Hernandez GM. Sensitive, high-throughput HLA-I and HLA-II immunopeptidomics using parallel accumulation-serial fragmentation mass spectrometry. Mol Cell Proteomics: MCP. (2023) 22:1005635. doi: 10.1016/j.mcpro.2023.100563 PMC10326702 37142057

[25] Choi S Paek E. pXg: comprehensive identification of noncanonical MHC-I-associated peptides from de novo peptide sequencing using RNA-seq reads. Mol Cell Proteomics: MCP. (2024) 23:1007435. doi: 10.1016/j.mcpro.2024.100743 PMC10979277 38403075

[26] Prensner JR Abelin JG Kok LW Clauser KR Mudge JM Ruiz-Orera J. What can ribo-seq, immunopeptidomics, and proteomics tell us about the noncanonical proteome? Molecular & Cellular Proteomics. (2023) 22(9):100631. doi: 10.1016/j.mcpro.2023.100631 37572790 PMC10506109

[27] Liao T-T Chen Y-H Li Z-Y Hsiao A-C Huang Y-L Hao R-X. Hypoxia-induced long non-coding RNA HIF1A−AS2 regulates stability of MHC class I protein in head and neck cancer. Cancer Immunol Res. (2024) 12(10):1468–84. doi: 10.1158/2326-6066.CIR-23-0622 PMC11443317 38920249

[28] Minegishi Y Haga Y Ueda K. Emerging potential of immunopeptidomics by mass spectrometry in cancer immunotherapy. Cancer Sci. (2024) 115:1048–595. doi: 10.1111/cas.16118 PMC11007014 38382459

[29] Hunt DF Henderson RA Shabanowitz J Sakaguchi K Michel H Sevilir N. Characterization of peptides bound to the class I MHC molecule HLA-A2.1 by mass spectrometry. Sci (New York N.Y.). (1992) 255:1261–63. doi: 10.1126/science.1546328 1546328

[30] Yang KL Yu F Teo GC Li K Demichev V Ralser M. MSBooster: improving peptide identification rates using deep learning-based features. Nat Commun. (2023) 14:45395. doi: 10.1038/s41467-023-40129-9 PMC10374903 37500632

[31] Kote S Pirog A Bedran G Alfaro J Dapic I. Mass spectrometry-based identification of MHC-associated peptides. Cancers. (2020) 12:5355. doi: 10.3390/cancers12030535 PMC7139412 32110973

[32] Mann M Kumar C Zeng W-F Strauss MT. Artificial intelligence for proteomics and biomarker discovery. Cell Syst. (2021) 12:759–05. doi: 10.1016/j.cels.2021.06.006 34411543

[33] Nesvizhskii AI. Proteogenomics: concepts, applications and computational strategies. Nat Methods. (2014) 11:1114–25. doi: 10.1038/nmeth.3144 PMC4392723 25357241

[34] Li R Ying B Liu Y Spencer JF Miao J Tollefson AE. Generation and characterization of an il2rg knockout Syrian hamster model for XSCID and HAdV-C6 infection in immunocompromised patients. Dis Models Mech. (2020) 13:dmm044602. doi: 10.1242/dmm.044602 PMC7473636 32651192

[35] Sturm T Sautter B Wörner TP Stevanović S Rammensee H-G Planz O. Mild acid elution and MHC immunoaffinity chromatography reveal similar albeit not identical profiles of the HLA class I immunopeptidome. J Proteome Res. (2021) 20:289–3045. doi: 10.1021/acs.jproteome.0c00386 33141586 PMC7786382

[36] Nicastri A Liao H Muller J Purcell AW Ternette N. The choice of HLA-associated peptide enrichment and purification strategy affects peptide yields and creates a bias in detected sequence repertoire. Proteomics. (2020) 20:e19004015. doi: 10.1002/pmic.201900401 32359108

[37] Zhang L McAlpine PL Heberling ML Joshua E. E. Automated ligand purification platform accelerates immunopeptidome analysis by mass spectrometry. J Proteome Res. (2021) 20:393–4085. doi: 10.1021/acs.jproteome.0c00464 33331781 PMC11391901

[38] Freudenmann LK Marcu A Stevanović S. Mapping the tumour human leukocyte antigen (HLA) ligandome by mass spectrometry. Immunology. (2018) 154:331–455. doi: 10.1111/imm.12936 29658117 PMC6002237

[39] Lorente E Marcilla M de la Sota PG Quijada-Freire A Mir C López D. Acid stripping after infection improves the detection of viral HLA class I natural ligands identified by mass spectrometry. Int J Mol Sci. (2021) 22:105035. doi: 10.3390/ijms221910503 PMC8508920 34638844

[40] Huisman BD Balivada PA Birnbaum ME. Yeast display platform with expression of linear peptide epitopes for high-throughput assessment of peptide-MHC-II binding. J Biol Chem. (2023) 299:1029135. doi: 10.1016/j.jbc.2023.102913 PMC9971316 36649909

[41] Andjus S Morillon A Wery M. From yeast to mammals, the nonsense-mediated mRNA decay as a master regulator of long non-coding RNAs functional trajectory. Non-Coding RNA. (2021) 7:445. doi: 10.3390/ncrna7030044 PMC8395947 34449682

[42] Wang H Meng Q Qian J Li M Gu C Yang Y. Review: RNA-based diagnostic markers discovery and therapeutic targets development in cancer. Pharmacol Ther. (2022) 234:108123. doi: 10.1016/j.pharmthera.2022.108123 35121000

[43] Crappé J Criekinge WV Trooskens G Hayakawa E Luyten W Baggerman G. Combining in silico prediction and ribosome profiling in a genome-wide search for novel putatively coding sORFs. BMC Genomics. (2013) 14:648. doi: 10.1186/1471-2164-14-648 24059539 PMC3852105

[44] Ingolia NT Brar GA Rouskin S McGeachy AM Weissman JS. Genome-wide annotation and quantitation of translation by ribosome profiling. Curr Protoc Mol Biol. (2013) 103:4.18.1–4.18.19. doi: 10.1002/0471142727.mb0418s103. Chapter 4. PMC3775365 23821443

[45] Bjerregaard A-M Nielsen M Jurtz V Barra CM Hadrup SR Szallasi Z. An analysis of natural T cell responses to predicted tumor neoepitopes. Front Immunol. (2017) 8:1566. doi: 10.3389/fimmu.2017.01566 29187854 PMC5694748

[46] Hansen K Kumar S Logronio K Whelan S Qurashi S Cheng H-Y. COM902, a novel therapeutic antibody targeting TIGIT augments anti-tumor T cell function in combination with PVRIG or PD-1 pathway blockade. Cancer Immunology Immunotherapy: CII. (2021) 70:3525–40. doi: 10.1007/s00262-021-02921-8 PMC10992303 33903974

[47] Raj A Wang SH Shim H Harpak A Li YI Engelmann B. Thousands of Novel Translated Open Reading Frames in Humans Inferred by Ribosome Footprint Profiling Vol. 5. Ingolia NT, editor. eLife (Cambridge, United Kingdom: eLife Sciences Publications, Ltd) (2016). doi: 10.7554/eLife.13328 PMC4940163 27232982

[48] Ji HJ Salzberg SL. Upstream open reading frames may contain hundreds of novel human exons. PloS Comput Biol. (2024) 20:e10125435. doi: 10.1371/journal.pcbi.1012543 PMC11578521 39565752

[49] Huang P Wen F Tuerhong N Yang Y Li Q. Neoantigens in cancer immunotherapy: focusing on alternative splicing. Front Immunol. (2024) 15:1437774. doi: 10.3389/fimmu.2024.1437774 39055714 PMC11269099

[50] Nagel R Pataskar A Champagne J Agami R. Boosting antitumor immunity with an expanded neoepitope landscape. Cancer Res. (2022) 82:3637–495. doi: 10.1158/0008-5472.CAN-22-1525 PMC9574376 35904353

[51] Szczepaniak A Bronisz A Godlewski J. Circular RNAs-new kids on the block in cancer pathophysiology and management. Cells. (2023) 12:5525. doi: 10.3390/cells12040552 PMC9953808 36831219

[52] Kacen A Javitt A Kramer MP Morgenstern D Tsaban T Shmueli MD. Post-translational modifications reshape the antigenic landscape of the MHC I immunopeptidome in tumors. Nat Biotechnol. (2023) 41:239–51. doi: 10.1038/s41587-022-01464-2 PMC11197725 36203013

[53] Zhao M Qu H. circVAR database: genome-wide archive of genetic variants for human circular RNAs. BMC Genomics. (2020) 21:7505. doi: 10.1186/s12864-020-07172-y PMC7599103 33121433

[54] Wu W Zhao F Zhang J. circAtlas 3.0: A gateway to 3 million curated vertebrate circular RNAs based on a standardized nomenclature scheme. Nucleic Acids Res. (2024) 52:D52–60. doi: 10.1093/nar/gkad770 PMC10767913 37739414

[55] Vita R Mahajan S Overton JA Dhanda SK Martini S Cantrell JR. The immune epitope database (IEDB): 2018 update. Nucleic Acids Res. (2019) 47:D339–43. doi: 10.1093/nar/gky1006 PMC6324067 30357391

[56] Volders P-J Helsens K Wang X Menten B Martens L Gevaert K. LNCipedia: A database for annotated human lncRNA transcript sequences and structures. Nucleic Acids Res. (2013) 41:D246–51. doi: 10.1093/nar/gks915 PMC3531107 23042674

[57] Glažar P Papavasileiou P Rajewsky N. circBase: A database for circular RNAs. RNA. (2014) 20:1666–70. doi: 10.1261/rna.043687.113 PMC4201819 25234927

[58] Huang X Gan Z Cui H Lan T Liu Y Caron E. The systeMHC atlas v2.0, an updated resource for mass spectrometry-based immunopeptidomics. Nucleic Acids Res. (2023) 52:D1062–71. doi: 10.1093/nar/gkad1068 PMC10767952 38000392

[59] Dragomir MP Manyam GC Ott LF Berland L Knutsen E Ivan C. FuncPEP: A database of functional peptides encoded by non-coding RNAs. Non-Coding RNA. (2020) 6:415. doi: 10.3390/ncrna6040041 PMC7712257 32977531

[60] Mathivanan S Fahner CJ Reid GE Simpson RJ. ExoCarta 2012: database of exosomal proteins, RNA and lipids. Nucleic Acids Res. (2012) 40:D1241–1244. doi: 10.1093/nar/gkr828 PMC3245025 21989406

[61] Chitti SV Gummadi S Kang T Shahi S Marzan AL Nedeva C. Vesiclepedia 2024: an extracellular vesicles and extracellular particles repository. Nucleic Acids Res. (2024) 52:D1694–98. doi: 10.1093/nar/gkad1007 PMC10767981 37953359

[62] Leblanc S Yala F Provencher N Lucier JF Levesque M Lapointe X. OpenProt 2.0 builds a path to the functional characterization of alternative proteins. Nucleic Acids Res. (2024) 52:D522–28. doi: 10.1093/nar/gkad1050 PMC10767855 37956315

[63] Luo X Huang Y Li H Luo Y Zuo Z Ren J. SPENCER: A comprehensive database for small peptides encoded by noncoding RNAs in cancer patients. Nucleic Acids Res. (2022) 50:D1373–81. doi: 10.1093/nar/gkab822 PMC8728293 34570216

[64] Liu H Zhou X Yuan M Zhou S Huang Y-E Hou F. ncEP: A manually curated database for experimentally validated ncRNA-encoded proteins or peptides. J Mol Biol. (2020) 432:3364–685. doi: 10.1016/j.jmb.2020.02.022 32105730

[65] Kang Y-J Yang D-C Kong L Hou M Meng Y-Q Wei L. CPC2: A fast and accurate coding potential calculator based on sequence intrinsic features. Nucleic Acids Res. (2017) 45:W12–16. doi: 10.1093/nar/gkx428 PMC5793834 28521017

[66] Calviello L Mukherjee N Wyler E Zauber H Hirsekorn A Selbach M. Detecting actively translated open reading frames in ribosome profiling data. Nat Methods. (2016) 13:165–705. doi: 10.1038/nmeth.3688 26657557

[67] Liu T Wu J Wu Y Hu W Fang Z Wang Z. LncPep: A resource of translational evidences for lncRNAs. Front Cell Dev Biol. (2022) 10:795084. doi: 10.3389/fcell.2022.795084 35141219 PMC8819059

[68] Perez-Riverol Y Csordas A Bai J Bernal-Llinares M Hewapathirana S Kundu DJ. The PRIDE database and related tools and resources in 2019: improving support for quantification data. Nucleic Acids Res. (2019) 47:D442–50. doi: 10.1093/nar/gky1106 PMC6323896 30395289

[69] Russo F Bella SD Nigita G Macca V Laganà A Giugno R. miRandola: extracellular circulating microRNAs database. PloS One. (2012) 7:e477865. doi: 10.1371/journal.pone.0047786 PMC3477145 23094086

[70] Ganesan K Kulandaisamy A Priya SB Gromiha MM. HuVarBase: A human variant database with comprehensive information at gene and protein levels. PloS One. (2019) 14:e02104755. doi: 10.1371/journal.pone.0210475 PMC6354970 30703169

[71] Alessio E Bonadio RS Buson L Chemello F Cagnin S. A Single Cell but Many Different Transcripts: A Journey into the World of Long Non-Coding RNAs. Int J Mol Sci. (2020) 21:3025. doi: 10.3390/ijms21010302 31906285 PMC6982300

[72] Ramakrishnaiah Y Kuhlmann L Tyagi S. Towards a comprehensive pipeline to identify and functionally annotate long noncoding RNA (lncRNA). Comput Biol Med. (2020) 127:104028. doi: 10.1016/j.compbiomed.2020.104028 33126123

[73] Bentum Mv Selbach M. An introduction to advanced targeted acquisition methods. Mol Cell Proteomics: MCP. (2021) 20:100165. doi: 10.1016/j.mcpro.2021.100165 34673283 PMC8600983

[74] Xie N Shen G Gao W Huang Z Huang C Fu L. Neoantigens: promising targets for cancer therapy. Signal Transduction Targeted Ther. (2023) 8:95. doi: 10.1038/s41392-022-01270-x PMC9816309 36604431

[75] Farge Dominique CF Connors JM Ay C Khorana AA Munoz A Brenner B. 2019 international clinical practice guidelines for the treatment and prophylaxis of venous thromboembolism in patients with cancer - pubMed. Lancet Oncol. (2019) 20:e566–81. doi: 10.1016/S1470-2045(19)30336-5 31492632

[76] Bathke J Konzer A Remes B McIntosh M Klug G. Comparative analyses of the variation of the transcriptome and proteome of rhodobacter sphaeroides throughout growth. BMC Genomics. (2019) 20:3585. doi: 10.1186/s12864-019-5749-3 PMC6509803 31072330

[77] Ambros V. microRNAs: tiny regulators with great potential. Cell. (2001) 107:823–26. doi: 10.1016/S0092-8674(01)00616-X 11779458

[78] Lee RC Feinbaum RL Ambros V. The C. Elegans heterochronic gene lin-4 encodes small RNAs with antisense complementarity to lin-14. Cell. (1993) 75:843–54. doi: 10.1016/0092-8674(93)90529-y 8252621

[79] Gauthier J Vincent AT Charette SJ Derome N. A brief history of bioinformatics. Briefings Bioinf. (2019) 20:1981–965. doi: 10.1093/bib/bby063 30084940

[80] Kute PM Soukarieh O Tjeldnes H Trégouët D-A Valen E. Small open reading frames, how to find them and determine their function. Front Genet. (2022) 12:796060. doi: 10.3389/fgene.2021.796060 35154250 PMC8831751

[81] Starck SR Shastri N. Non-conventional sources of peptides presented by MHC class I. Cell Mol Life Sci. (2011) 68:1471–795. doi: 10.1007/s00018-011-0655-0 PMC3071930 21390547

[82] Su D Ding C Qiu J Yang G Wang R Liu Y. Ribosome profiling: A powerful tool in oncological research. biomark Res. (2024) 12:115. doi: 10.1186/s40364-024-00562-4 38273337 PMC10809610

[83] Tian H Tang L Yang Z Xiang Y Min Q Yin M. Current understanding of functional peptides encoded by lncRNA in cancer. Cancer Cell Int. (2024) 24:2525. doi: 10.1186/s12935-024-03446-7 PMC11265036 39030557

[84] Wei J Li M Xue C Chen S Zheng L Deng H. Understanding the roles and regulation patterns of circRNA on its host gene in tumorigenesis and tumor progression. J Exp Clin Cancer Res. (2023) 42:86. doi: 10.1186/s13046-023-02657-6 37060016 PMC10105446

[85] Huang D Zhu X Ye S Zhang J Liao J Zhang N. Tumour circular RNAs elicit anti-tumour immunity by encoding cryptic peptides. Nature. (2024) 625:593–602. doi: 10.1038/s41586-023-06834-7 38093017

[86] Walkley CR Li JB. Rewriting the transcriptome: adenosine-to-inosine RNA editing by ADARs. Genome Biol. (2017) 18:2055. doi: 10.1186/s13059-017-1347-3 PMC5663115 29084589

[87] Kwok DW Okada H Costello JF. Activating the dark genome to illuminate cancer vaccine targets. Nat Genet. (2024) 56:1770–715. doi: 10.1038/s41588-024-01850-3 PMC11456370 39223317

[88] Zhang B Bassani-Sternberg M. Current perspectives on mass spectrometry-based immunopeptidomics: the computational angle to tumor antigen discovery. J ImmunoTherapy Cancer. (2023) 11:e0070735. doi: 10.1136/jitc-2023-007073 PMC10619091 37899131

[89] Zhang T Kurban E Wang Z. Neoantigens: the novel precision cancer immunotherapy. Biologics. (2023) 3:321–45. doi: 10.3390/biologics3040017

[90] Yesavage T. Advances in immunopeptidomics. Biocompare. (2024). https://www.biocompare.com/Editorial-Articles/611366-Advances-in-Immunopeptidomics/ (Accessed September 15, 2024).

[91] Li X Pak HS Huber F Michaux J Taillandier-Coindard M Altimiras ER. A microfluidics-enabled automated workflow of sample preparation for MS-based immunopeptidomics. Cell Rep Methods. (2023) 3:1004795. doi: 10.1016/j.crmeth.2023.100479 PMC10326370 37426762

[92] Gfeller D Liu Y Racle J. Contemplating immunopeptidomes to better predict them. Semin Immunol. (2023) 66:101708. doi: 10.1016/j.smim.2022.101708 36621290

[93] Pu T Peddle A Zhu J Tejpar S Verbandt S. Chapter 9 - Neoantigen Identification: Technological Advances and Challenges. In: Garg A Galluzzi L, editors. Methods in Cell Biology, vol. 183. Cambridge, Massachusetts, United States: Academic Press (2024). p. 265–302. doi: 10.1016/bs.mcb.2023.06.005 38548414

[94] Ferreira HJ Stevenson BJ Pak H Yu F Oliveira JA Huber F. Immunopeptidomics-based identification of naturally presented non-canonical circRNA-derived peptides. Nat Commun. (2024) 15:2357. doi: 10.1038/s41467-024-46408-3 38490980 PMC10943130

[95] Zhang Z Yan C Li K Bao S Li L Chen L. Pan-Cancer Characterization of lncRNA Modifiers of Immune Microenvironment Reveals Clinically Distinct de Novo Tumor Subtypes. NPJ Genomic Med. (2021) 6:1–115. doi: 10.1038/s41525-021-00215-7 PMC8211863 34140519

[96] Chen B Dragomir MP Yang C Li Q Horst D Calin GA. Targeting non-coding RNAs to overcome cancer therapy resistance. Signal Transduction Targeted Ther. (2022) 7:1–205. doi: 10.1038/s41392-022-00975-3 PMC9008121 35418578

[97] Shiu PKT Ilieva M Holm A Uchida S DiStefano JK Bronisz A. The non-coding RNA journal club: highlights on recent papers-12. Non-Coding RNA. (2023) 9:28. doi: 10.3390/ncrna9020028 37104010 PMC10144170

[98] Li J Xiao Z Wang D Jia L Nie S Zeng X. The screening, identification, design and clinical application of tumor-specific neoantigens for TCR-T cells. Mol Cancer. (2023) 22:1415. doi: 10.1186/s12943-023-01844-5 PMC10466891 37649123

[99] Chakraborty C Majumder A Bhattacharya M Chatterjee S Lee S-S. The landscape of neoantigens and its clinical applications: from immunobiology to cancer vaccines. Curr Res Biotechnol. (2024) 7:100177. doi: 10.1016/j.crbiot.2024.100177

[100] Kim SH Lee BR Kim S-M Kim S Kim M-s Kim J. The identification of effective tumor-suppressing neoantigens using a tumor-reactive TIL TCR-pMHC ternary complex. Exp Mol Med. (2024) 56:1461–71. doi: 10.1038/s12276-024-01259-2 PMC11263684 38866910

[101] Li L Goedegebuure SP Gillanders WE. Preclinical and clinical development of neoantigen vaccines. Ann Oncology: Off J Eur Soc Med Oncol. (2017) 28:xii11–17. doi: 10.1093/annonc/mdx681 PMC5834106 29253113

[102] Alahdal M Eyad E. Non-coding RNAs in cancer immunotherapy: predictive biomarkers and targets. Clin Trans Med. (2023) 13:e14255. doi: 10.1002/ctm2.1425 PMC10514379 37735815

[103] Diao L Liu M. Rethinking antigen source: cancer vaccines based on whole tumor cell/tissue lysate or whole tumor cell. Advanced Sci. (2023) 10:23001215. doi: 10.1002/advs.202300121 PMC10401146 37254712

[104] Biri-Kovács B Bánóczi Z Tummalapally A Szabó I. Peptide vaccines in melanoma: chemical approaches towards improved immunotherapeutic efficacy. Pharmaceutics. (2023) 15:4525. doi: 10.3390/pharmaceutics15020452 PMC9963291 36839774

[105] Ricklefs FL Alayo Q Krenzlin H Mahmoud AB Speranza MC Nakashima H. Immune evasion mediated by PD-L1 on glioblastoma-derived extracellular vesicles. Sci Adv. (2018) 4:eaar2766. doi: 10.1126/sciadv.aar2766 29532035 PMC5842038

[106] Castillo-Peña A Molina-Pinelo S. Landscape of tumor and immune system cells-derived exosomes in lung cancer: mediators of antitumor immunity regulation. Front Immunol. (2023) 14:1279495. doi: 10.3389/fimmu.2023.1279495 37915578 PMC10616833

[107] Liu H Chen L Peng Y Yu S Liu J Wu L. Dendritic cells loaded with tumor derived exosomes for cancer immunotherapy. Oncotarget. (2017) 9:2887–94. doi: 10.18632/oncotarget.20812 PMC5788689 29416821

[108] Jung I Shin S Baek M-C Yea K. Modification of immune cell-derived exosomes for enhanced cancer immunotherapy: current advances and therapeutic applications. Exp Mol Med. (2024) 56:19–315. doi: 10.1038/s12276-023-01132-8 38172594 PMC10834411

[109] Bartel DP. MicroRNAs: genomics, biogenesis, mechanism, and function. Cell. (2004) 116:281–97. doi: 10.1016/S0092-8674(04)00045-5 14744438

[110] Fire A Xu S Montgomery MK Kostas SA Driver SE Mello CC. Potent and specific genetic interference by double-stranded RNA in caenorhabditis elegans. Nature. (1998) 391:806–11. doi: 10.1038/35888 9486653

[111] Laumont CM Vincent K Hesnard L Audemard É Bonneil É Laverdure J-P. Noncoding regions are the main source of targetable tumor-specific antigens. Sci Trans Med. (2018) 10:eaau5516. doi: 10.1126/scitranslmed.aau5516 30518613

[112] Kondo T Plaza S Zanet J Benrabah E Valenti P Hashimoto Y. Small peptides switch the transcriptional activity of shavenbaby during drosophila embryogenesis. Science. (2010) 329:336–39. doi: 10.1126/science.1188158 20647469

[113] Laumont CM Daouda T Laverdure J-P Bonneil É Caron-Lizotte O Hardy M-P. Global proteogenomic analysis of human MHC class I-associated peptides derived from non-canonical reading frames. Nat Commun. (2016) 7:10238. doi: 10.1038/ncomms10238 26728094 PMC4728431

[114] Kuznetsov A Voronina A Govorun V Arapidi G. Critical review of existing MHC I immunopeptidome isolation methods. Molecules. (2020) 25:54095. doi: 10.3390/molecules25225409 PMC7699222 33228004

[115] Chloe Chong MM Pak H Harnett D Huber F Grun D Leleu M. Integrated proteogenomic deep sequencing and analytics accurately identify non-canonical peptides in tumor immunopeptidomes - pubMed. Nat Commun. (2020) 11:1293. doi: 10.1038/s41467-020-14968-9 32157095 PMC7064602

[116] Chemali M Radtke K Desjardins M English L. Alternative pathways for MHC class I presentation: A new function for autophagy. Cell Mol Life Sciences: CMLS. (2011) 68:1533–415. doi: 10.1007/s00018-011-0660-3 PMC11114914 21390546

[117] Sijts EJAM Kloetzel P-M. The role of the proteasome in the generation of MHC class I ligands and immune responses. Cell Mol Life Sciences: CMLS. (2011) 68:14915. doi: 10.1007/s00018-011-0657-y PMC3071949 21387144

[118] Kumar MA Baba SK Sadida HQ Marzooqi SA Jerobin J Altemani FH. Extracellular vesicles as tools and targets in therapy for diseases. Signal Transduction Targeted Ther. (2024) 9:1–41. doi: 10.1038/s41392-024-01735-1 PMC10838959 38311623

[119] Zhang Y Liang F Zhang D Qi S Liu Y. Metabolites as extracellular vesicle cargo in health, cancer, pleural effusion, and cardiovascular diseases: an emerging field of study to diagnostic and therapeutic purposes. Biomedicine Pharmacotherapy. (2023) 157:114046. doi: 10.1016/j.biopha.2022.114046 36469967

[120] Matsuzaka Y Yashiro R. Regulation of extracellular vesicle-mediated immune responses against antigen-specific presentation. Vaccines. (2022) 10:16915. doi: 10.3390/vaccines10101691 PMC9607341 36298556

[121] Neisig A Melief CJM Neefjes J. Reduced cell surface expression of HLA-C molecules correlates with restricted peptide binding and stable TAP interaction1. J Immunol. (1998) 160:171–795. doi: 10.4049/jimmunol.160.1.171 9551969

[122] Howard JC. Supply and transport of peptides presented by class I MHC molecules. Curr Opin Immunol. (1995) 7:69–76. doi: 10.1016/0952-7915(95)80031-X 7772284

[123] Milner E Gutter-Kapon L Bassani-Strenberg M Barnea E Beer I Admon A. The effect of proteasome inhibition on the generation of the human leukocyte antigen (HLA) peptidome. Mol Cell Proteomics: MCP. (2013) 12:1853–645. doi: 10.1074/mcp.M112.026013 PMC3708171 23538226

[124] Shi S Ou X Liu C Wen H Jiang K. Immunoproteasome acted as immunotherapy ‘Coffee companion’ in advanced carcinoma therapy. Front Immunol. (2024) 15:1464267. doi: 10.3389/fimmu.2024.1464267 39281672 PMC11392738

[125] Ferrington DA Gregerson DS. Chapter 3 - Immunoproteasomes: Structure, Function, and Antigen Presentation. In: Grune T, editor. Progress in Molecular Biology and Translational Science, vol. 109. Cambridge, Massachusetts, United States: Academic Press (2012). p. 75–112. doi: 10.1016/B978-0-12-397863-9.00003-1 PMC4405001 22727420

[126] Pongcharoen S Kaewsringam N Somaparn P Roytrakul S Maneerat Y Pintha K. Immunopeptidomics in the cancer immunotherapy era. Explor Targeted Anti-Tumor Ther. (2024) 5:801–175. doi: 10.37349/etat.2024.00249 PMC11390293 39280250

[127] Dean P Heunis T Härtlova A Trost M. Regulation of phagosome functions by post-translational modifications: A new paradigm. Curr Opin Chem Biology Omics. (2019) 48:73–80. doi: 10.1016/j.cbpa.2018.11.001 30481638

[128] Bilkei-Gorzo O Heunis T Marín-Rubio JL Cianfanelli FR Raymond BBA Inns J. The E3 ubiquitin ligase RNF115 regulates phagosome maturation and host response to bacterial infection. EMBO J. (2022) 41:e108970. doi: 10.15252/embj.2021108970 36281581 PMC9713710

[129] Xia H Green DR Zou W. Autophagy in tumour immunity and therapy. Nat Rev Cancer. (2021) 21:281–975. doi: 10.1038/s41568-021-00344-2 33758415 PMC8087647

[130] Roche PA Furuta K. The ins and outs of MHC class II-mediated antigen processing and presentation. Nat Rev Immunol. (2015) 15:203–165. doi: 10.1038/nri3818 25720354 PMC6314495

[131] Blachère NE Darnell RB Albert ML. Apoptotic cells deliver processed antigen to dendritic cells for cross-presentation. PloS Biol. (2005) 3:e1855. doi: 10.1371/journal.pbio.0030185 PMC1084338 15839733

[132] Yang J Liu M Fang X Zhang H Ren Q Zheng Y. Advances in peptides encoded by non-coding RNAs: A cargo in exosome. Front Oncol. (2022) 12:1081997. doi: 10.3389/fonc.2022.1081997 36620552 PMC9822543

[133] Hamieh M Dobrin A Cabriolu A Stegen SJCv Giavridis T Mansilla-Soto J. CAR T cell trogocytosis and cooperative killing regulate tumour antigen escape. Nature. (2019) 568:112–16. doi: 10.1038/s41586-019-1054-1 PMC6707377 30918399

[134] Saccheri F Pozzi C Avogadri F Barozzi S Faretta M Fusi P. Bacteria-induced gap junctions in tumors favor antigen cross-presentation and antitumor immunity. Sci Trans Med. (2010) 2:44ra57–7. doi: 10.1126/scitranslmed.3000739 20702856

[135] Antón LC Yewdell JW. Translating DRiPs: MHC class I immunosurveillance of pathogens and tumors. J Leukocyte Biol. (2014) 95:551–625. doi: 10.1189/jlb.1113599 24532645 PMC3958739

[136] Wojtukiewicz MZ Rek MM Karpowicz K Górska M Polityńska B Wojtukiewicz AM. Inhibitors of immune checkpoints—PD-1, PD-L1, CTLA-4—New opportunities for cancer patients and a new challenge for internists and general practitioners. Cancer Metastasis Rev. (2021) 40:949–825. doi: 10.1007/s10555-021-09976-0 34236546 PMC8556173

[137] Grivennikov SI Greten FR Karin M. Immunity, inflammation, and cancer. Cell. (2010) 140:883–995. doi: 10.1016/j.cell.2010.01.025 20303878 PMC2866629

[138] Liu L Wang Q Qiu Z Kang Y Liu J Ning S. Noncoding RNAs: the shot callers in tumor immune escape. Signal Transduction Targeted Ther. (2020) 5:1–245. doi: 10.1038/s41392-020-0194-y PMC7305134 32561709

[139] Zhong Q Xiao X Qiu Y Xu Z Chen C Chong B. Protein posttranslational modifications in health and diseases: functions, regulatory mechanisms, and therapeutic implications. MedComm. (2023) 4:e261. doi: 10.1002/mco2.261 37143582 PMC10152985

[140] Utz PJ Hottelet M Schur PH Anderson P. Proteins phosphorylated during stress-induced apoptosis are common targets for autoantibody production in patients with systemic lupus erythematosus. J Exp Med. (1997) 185:843–545. doi: 10.1084/jem.185.5.843 9120390 PMC2196161

[141] Rock KL Farfán-Arribas DJ Colbert JD Goldberg AL. MHC class I-presented peptides and the DRiP hypothesis. Trends Immunol. (2014) 35:144–525. doi: 10.1016/j.it.2014.01.002 24566257 PMC3986829

[142] Yewdell JW. DRiPs solidify: progress in understanding endogenous MHC class I antigen processing. Trends Immunol. (2011) 32:548–58. doi: 10.1016/j.it.2011.08.001 PMC3200450 21962745

[143] Ramalho S Dopler A Faller WJ. Ribosome specialization in cancer: A spotlight on ribosomal proteins. NAR Cancer. (2024) 6:zcae029. doi: 10.1093/narcan/zcae029 38989007 PMC11231584

[144] Todaro B Ottalagana E Luin S Santi M. Targeting peptides: the new generation of targeted drug delivery systems. Pharmaceutics. (2023) 15:16485. doi: 10.3390/pharmaceutics15061648 PMC10300729 37376097

[145] Rana PS Ignatz-Hoover JJ Driscoll JJ. Targeting proteasomes and the MHC class I antigen presentation machinery to treat cancer, infections and age-related diseases. Cancers. (2023) 15:56325. doi: 10.3390/cancers15235632 PMC10705104 38067336

[146] Truong HV Sgourakis NG. Dynamics of MHC-I molecules in the antigen processing and presentation pathway. Curr Opin Immunol. (2021) 70:122–28. doi: 10.1016/j.coi.2021.04.012 PMC8622473 34153556

[147] Cruz FM Colbert JD Merino E Kriegsman BA Rock KL. The biology and underlying mechanisms of cross-presentation of exogenous antigens on MHC I molecules. Annu Rev Immunol. (2017) 35:149–76. doi: 10.1146/annurev-immunol-041015-055254 PMC5508990 28125356

[148] Crotzer VL Blum JS. Autophagy and its role in MHC-mediated antigen presentation. J Immunol (Baltimore Md. : 1950). (2009) 182:3335–415. doi: 10.4049/jimmunol.0803458 PMC2730830 19265109

[149] Rock KL Farfán-Arribas DJ Shen L. Proteases in MHC class I presentation and cross-presentation. J Immunol (Baltimore Md. : 1950). (2010) 184:9–155. doi: 10.4049/jimmunol.0903399 PMC3094101 20028659

[150] Zhang J Luo Q Li X Guo J Zhu Q Lu X. Novel role of immune-related non-coding RNAs as potential biomarkers regulating tumour immunoresponse *via* MICA/NKG2D pathway. biomark Res. (2023) 11:86. doi: 10.1186/s40364-023-00530-4 37784183 PMC10546648

[151] Della Bella E Koch J Baerenfaller K. Translation and emerging functions of non-coding RNAs in inflammation and immunity. Allergy. (2022) 77:2025–375. doi: 10.1111/all.15234 PMC9302665 35094406

[152] Pi Y-N Qi W-C Xia B-R Lou G Jin W-L. Long non-coding RNAs in the tumor immune microenvironment: biological properties and therapeutic potential. Front Immunol. (2021) 12:697083. doi: 10.3389/fimmu.2021.697083 34295338 PMC8290853

[153] Godlewski J Farhath M Ricklefs FL Passaro C Kiel K Nakashima H. Oncolytic virus therapy alters the secretome of targeted glioblastoma cells. Cancers. (2021) 13:12875. doi: 10.3390/cancers13061287 PMC7999647 33799381

[154] Marar C Starich B Wirtz D. Extracellular vesicles in immunomodulation and tumor progression. Nat Immunol. (2021) 22:560–705. doi: 10.1038/s41590-021-00899-0 33753940 PMC9389600

[155] Arrè V Mastrogiacomo R Balestra F Serino G Viti F Rizzi F. Unveiling the potential of extracellular vesicles as biomarkers and therapeutic nanotools for gastrointestinal diseases. Pharmaceutics. (2024) 16:5675. doi: 10.3390/pharmaceutics16040567 PMC11055174 38675228

[156] Rock KL Reits E Neefjes J. Present yourself! By MHC class I and MHC class II molecules. Trends Immunol. (2016) 37:724–375. doi: 10.1016/j.it.2016.08.010 27614798 PMC5159193

[157] Kawashima M Higuchi H Kotani A. Significance of trogocytosis and exosome-mediated transport in establishing and maintaining the tumor microenvironment in lymphoid Malignancies. J Clin Exp Hematopathology : JCEH. (2021) 61:192–2015. doi: 10.3960/jslrt.21005 PMC8808107 34193756

[158] Admon A. The Biogenesis of the Immunopeptidome. In: Seminars in Immunology (Amsterdam, Netherlands: Elsevier Ltd), vol. 67. (2023). p. 101766. doi: 10.1016/j.smim.2023.101766 37141766

[159] Dixson A Dawson TR Vizio DD Weaver AM. Context-specific regulation of extracellular vesicle biogenesis and cargo selection. Nat Rev Mol Cell Biol. (2023) 24:454–765. doi: 10.1038/s41580-023-00576-0 36765164 PMC10330318

[160] Zong Y Wang Y Qiu X Huang X Qiao L. Deep learning prediction of glycopeptide tandem mass spectra powers glycoproteomics. Nat Mach Intell. (2024) 6:950–615. doi: 10.1038/s42256-024-00875-x

[161] Yang Y Liu X Shen C Lin Y Yang P Qiao L. In silico spectral libraries by deep learning facilitate data-independent acquisition proteomics. Nat Commun. (2020) 11:1465. doi: 10.1038/s41467-019-13866-z 31919359 PMC6952453

[162] Callaway E. Chemistry nobel goes to developers of alphaFold AI that predicts protein structures. Nature. (2024) 634:525–26. doi: 10.1038/d41586-024-03214-7 39384918

[163] Vrunda Trivedi CY Klippel K Yegorov O Roemeling Cv Hoang-Minh L Fenton G. mRNA-based precision targeting of neoantigens and tumor-associated antigens in Malignant brain tumors - pubMed. Genome Med. (2024) 16:17. doi: 10.1186/s13073-024-01281-z 38268001 PMC10809449

